# Supplementary material for: Heterocyclic sterol probes for live monitoring of sterol trafficking and lysosomal storage disorders
Source: Sci Rep. 2018 Sep 26;8:14428. doi: 10.1038/s41598-018-32776-6 (PMC6158244; doi:10.1038/s41598-018-32776-6)
Supplement: Supplementary file 1 — Supplementary Information [file 41598_2018_32776_MOESM1_ESM.pdf]

Supplementary Information

Heterocyclic sterol probes for live monitoring of sterol trafficking and lysosomal storage disorders

Jarmila Králová*, Michal Jurášek, Lucie Krčová, Bohumil Dolenský, Ivan Novotný, Michal Dušek, Zdeňka Rottnerová, Michal Kahle, Pavel Drašar, Petr Bartůněk, and Vladimír Král

*To whom correspondence may be addressed: kralova@img.cas.cz

Table of Contents:

1. Chemistry (Figs. S1-S3) 2

   1.1. Synthesis 3

   1.2. Compounds characterization (Table S1) 12

2. Crystallography (Figs. S4 and S5, Tables S2 and S3) 13

3. Spectral analysis (Fig. S6 and S7, Tables S4 - S6) 19

4. Cellular studies (Figs. S8-S15) 23

5. Movie Legends 30

6. References 31

1. Chemistry

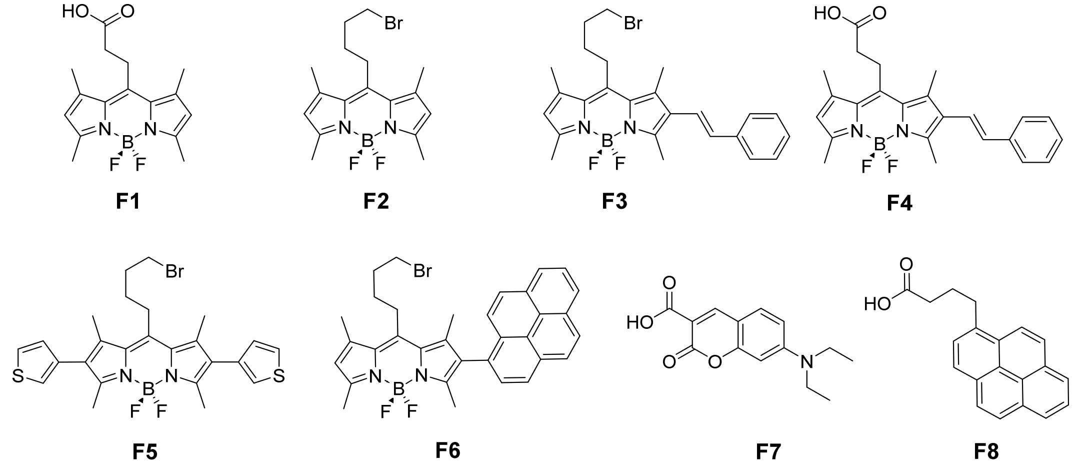

Fig. S1. Fluorophores used for synthesis of sterol probes

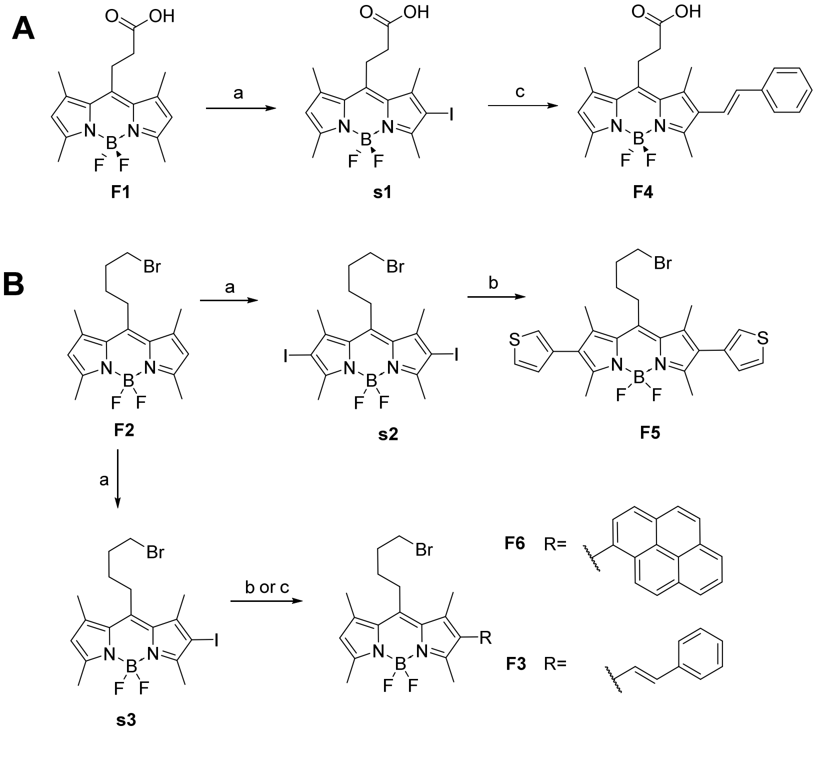

Fig. S2. Synthesis of BODIPY flurophores. *Reagents and conditions*: a) *N*-iodosuccinimide, DCM, RT; b) $Pd(PPh_3)_4$, Na_2CO_3, toluene-MeOH-H_2O, thienylboronic acid or pyrene-1-boronic acid, 80 °C 14 h; c) $Pd(dppf)_2Cl_2$, Na_2CO_3, AcCN, *trans*-2-phenylvinylboronic acid, 80 °C, 14 h.

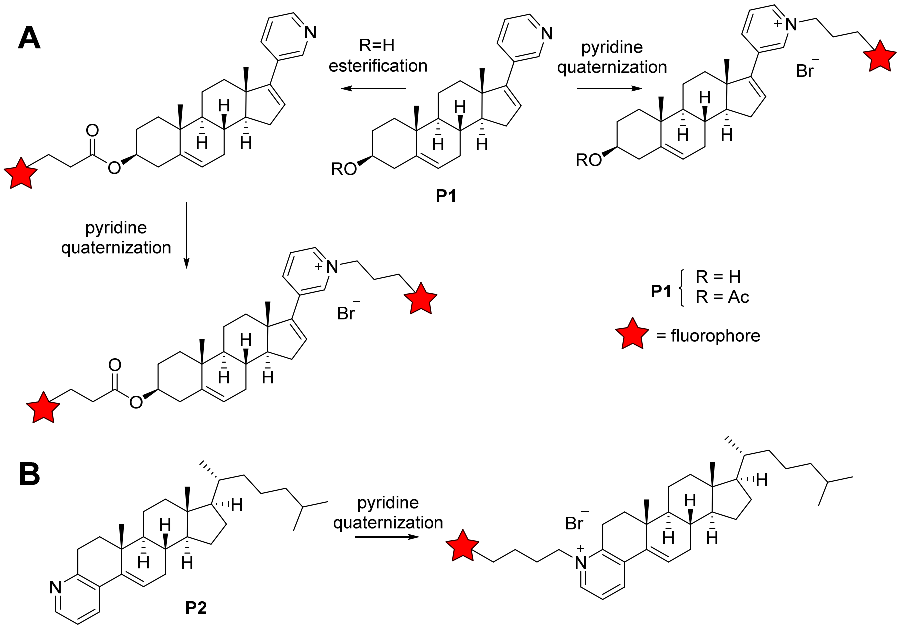

Fig. S3. General methods for derivatization of precursors **P1 (A)** and **P2 (B)** by fluorophores **F1 - F8**. Esterification was conducted using Steglich protocol using either DCC or EDCI as coupling agent. Quaternization was performed by refluxing a mixture of **P1** or **P2** and appropriate fluorescent alkyl bromide in AcCN.

1.1. Synthesis

BODIPY s1: To a solution of BODIPY **F1**[1] (267 mg, 0.83 mmol) in dry DCM (50 mL) NIS (187 mg, 0.83 mmol) was added and the mixture was stirred at RT for 1 h. Then the solvent was evaporated under reduced pressure and the residue chromatographed (DCM/MeOH, 100/1 → 50/1) to obtain product **s1** (286 mg, 0.64 mmol) as red solids in 77% yield. R_F=0.7 in AcOEt. ^{1}H NMR (300 MHz, $CDCl_3$): 8.80 (1H, br s), 6.15 (1H, s), 3.42-3.30 (2H, m), 2.72-2.63 (2H, m), 2.78 (3H, s), 2.61 (3H, s), 2.54 (2H, s), 2.50 (3H, s), 2.47 (3H, s). ^{13}C{^{1}H} NMR (75 MHz, $CDCl_3$): 177.96, 176.15, 157.14, 154.10, 142.54, 140.18, 123.20, 110.00, 86.61, 34.88, 29.52, 23.77, 18.64, 16.70, 16.00, 14.73. HRMS-ESI: *exact mass* 446.04741 Da, found *m/z* 469.03666 $[M+Na]^+$ and 485.01810 $[M+K]^+$.

BODIPY F4: To a solution of BODIPY **s1** (500 mg, 1.12 mmol), *trans*-2-phenylvinylboronic acid (663 mg, 4.5 mmol) and Na_2CO_3 (475 mg, 4.5 mmol) in AcCN (25 mL) $Pd(dppf)_2Cl_2$ (88 mg, 0.12 mmol) was added. The mixture was stirred overnight at 60°C. Heating was removed and the solvents were evaporated under reduced pressure. The residue was diluted with water, acidified by HCl solution (0.1 M) and extracted with AcOEt. Combined organic layers were dried over Na_2SO_4, filtered and the solvents were evaporated under reduced pressure. Residue thus obtained was chromatographed (chloroform/MeOH, 100/1). The product **F4** (241 mg, 0.57 mmol) was obtained as red solids in 51% yield. R_F=0.5 in DCM-MeOH, 20/1. ^{1}H NMR (300 MHz, $CDCl_3$): 7.48 (2H, br d, 7.8), 7.37 (2H, br t, 7.6), 7.27 (1H, tt, 7.3, 1.2), 6.94 (1H, d, 16.5), 6.69 (1H, d, 16.5), 6.10 (1H, s), 3.40 (2H, br m), 2.70 (2H, m), 2.68 (3H, s), 2.55 (3H, s), 2.53 (3H, s), 2.47 (3H, s). ^{13}C{^{1}H} NMR (75 MHz, $CDCl_3$): 177.33, 155.09, 154.28, 142.48, 140.44, 137.52, 135.88, 132.49, 131.51, 130.93, 129.28, 128.72, 127.65, 126.17, 122.31, 119.55, 35.13, 23.46, 16.57, 14.54 (t, 2.2), 14.25, 13.95 (t, 3.0). HRMS-ESI: *exact mass* 422.19771 Da, found *m/z* 445.18686 $[M+Na]^+$.

BODIPY s2: This compound was synthesized similarly to BODIPY **s1** with BODIPY **F2**[2] and 4 eqvivalents of NIS. After column chromatography in hexanes-DCM (3/2, resp.) and evaporation of solvents the product was re-dissolved in small amount of chloroform, precipitated by hexanes and collected by filtration. Product **s2** was obtained as red solids in 78% yield. R_F=0.8 in hexanes-AcOEt, 4/1. ^{1}H NMR (400 MHz, $CDCl_3$): 3.46 (2H, t, 6.3), 3.07-2.99 (2H, m), 2.63 (6H, s), 2.49 (6H, m), 2.12-2.03 (2H, m), 1.85-1.75 (2H, m). $^{13}C\{^1H\}$ NMR (101 MHz, $CDCl_3$): 155.59, 144.93, 142.16, 131.31, 86.63, 32.90, 32.54, 29.88, 28.34, 19.02, 16.15 (t, 2.7). HRMS-ESI: *exact mass* 633.89603 Da, found *m/z* 632.88904 $[M-H]^-$.

BODIPY F5: To a solution of BODIPY **s2** (427 mg, 0.67 mmol) and 3-thienylboronic acid (344 mg, 2.7 mmol) in toluene-MeOH (7/3, 10 mL) was added aqueous Na_2CO_3 (213 mg, 2 mmol in 1 mL) and the mixture was stirred for 10 minutes after which $Pd(PPh_3)_4$ (154 mg, 0.13 mmol) was added. The mixture was stirred 12 h at RT and then additional Pd catalyst (154 mg) was added. The temperature was raised to 80 °C and stirring was continued for the next 6 h. The heating was removed and the mixture was diluted with AcOEt (50 mL) and washed with HCl solution (3×100 mL, 0.1 M) and water. Organic layer was dried over Na_2SO_4, filtered and the solvents were removed under reduced pressure. The residue was chromatographed (hexanes-DCM, 3/2). Material thus obtained was re-dissolved in small amount of DCM and precipitated by the addition of hexanes and filtered. Product **F5** (266 mg, 0.49 mmol) was obtained as orange solids in 73% yield. R_F=0.4 in hexanes-DCM, 3/2. ^{1}H NMR (400 MHz, $CDCl_3$): 7.42 (2H, dd, 4.9, 3.0), 7.26 (2H, s), 7.14 (2H, dd, 3.0, 1.3), 7.03 (2H, dd, 4.9, 1.3), 3.46 (2H, t, 6.5), 3.12 (2H, m, ΣJ = 17.1), 2.52 (3H, s), 2.39 (3H, s), 2.08 (2H, br kv, 7.1), 1.88 (2H, br m). $^{13}C\{^1H\}$ NMR (101 MHz, $CDCl_3$): 153.41, 145.71, 136.90, 133.84, 131.46, 129.45, 129.05, 125.62, 124.20, 77.16, 33.21, 32.90, 30.36, 27.96, 14.68, 13.53 (t, 2.8). HRMS-ESI: *exact mass* 546.07819 Da, found *m/z* 545.07098 $[M-H]^-$.

BODIPY s3: Synthesized similarly to BODIPY **s1** from BODIPY **F2**. After column chromatography in hexanes-DCM (3/2, resp.) and evaporation of solvents the product was re-dissolved in small amount of chloroform, precipitated by hexanes and collected by filtration. Product **s3** was obtained as red solids in 79% yield. R_F=0.6 in hexanes-AcOEt, 5/1. ^{1}H NMR (400 MHz, $CDCl_3$): 6.13 (1H, s), 3.45 (2H, t, 6.3), 3.00-2.92 (2H, m), 2.61 (3H, s), 2.53 (3H, s), 2.45 (3H, s), 2.42 (3H, s), 2.05 (2H, quin, 7.1), 1.84-1.72 (2H, m). $^{13}C\{^1H\}$ NMR (101 MHz, $CDCl_3$): 156.33, 153.34, 145.11, 142.37, 140.05, 131.85, 130.87, 122.89, 85.15, 32.96, 32.61, 29.95, 27.84, 18.57, 16.67, 15.90 (t, 2.3), 14.63 (t, 2.3). HRMS-ESI: *exact mass* 507.99939 Da, found *m/z* 530.98871 $[M+Na]^+$ and 546.96259 $[M+K]^+$.

BODIPY F3: To a solution of BODIPY **s3** (340 mg, 0.67 mmol), *trans*-2-phenylvinylboronic acid (395 mg, 2.7 mmol) in AcCN (10 mL) was added aqueous solution of Na_2CO_3 (286 mg, 2.7 mmol in 1.5 mL). The mixture was stirred at RT for 10 minutes after which $Pd(dppf)_2Cl_2$ (102 mg, 0.14 mmol) was added and the mixture was stirred for 14 h at 80 °C. Then it was cooled to RT, diluted with AcOEt (50 mL) and washed with HCl solution (3×100 mL, 0.1 M) and water. Organic layer was dried over Na_2SO_4, filtered and the solvents were removed under reduced pressure. The residue was chromatographed (hexanes-DCM, 3/2). Material thus obtained was re-dissolved in small amount of AcOEt and precipitated by the addition of hexanes. Product **F3** (253 mg, 0.52 mmol) was collected by filtration as red solids in 78% yield. ^{1}H NMR (300 MHz, $CDCl_3$): 7.47 (2H, br d, 7.8), 7.36 (2H, br t, 7.5), 7.26 (1H, tt, 7.4, 1.2), 6.95 (1H, d, 16.5), 6.68 (1H, d, 16.5), 6.09 (1H, s), 3.46 (2H, t, 6.4), 3.04 (2H, br m), 2.67 (3H, s), 2.53 (3H, s), 2.50 (3H, s), 2.44 (3H, s), 2.07 (2H, tt, 7.7, 7.4), 1.83 (2H, m). $^{13}C\{^1H\}$ NMR (75 MHz, $CDCl_3$): 154.52, 153.63, 145.31, 140.52, 137.79, 136.06, 132.35, 131.88, 131.27, 129.08, 128.86, 127.71, 126.31, 122.20, 119.95, 77.16, 33.20, 32.88, 30.33, 27.79, 16.77, 14.67 (t, 2.5), 14.42, 14.07 (t, 3.2). HRMS-ESI: *exact mass* 484.14970 Da, found *m/z* 507.13901 $[M+Na]^+$ and 523.11267 $[M+K]^+$.

BODIPY F6: To a solution of BODIPY **s3** (180 mg, 0.35 mmol) and pyrene-1-boronic acid (191 mg, 0.78 mmol) in toluene-MeOH (7/3, 10 mL) was added Na_2CO_3 (117 mg, 1.1 mmol) and $Pd(PPh_3)_4$ (81 mg, 71 μmol). The mixture was stirred at 70 °C for 14 h. Then the mixture was extracted with AcOEt, washed with HCl (0.1 M, 2×100 mL) and water (2×100 mL). Organic phase was dried over Na_2SO_4 and filtered. Solvents were evaporated under reduced pressure and the residue chromatographed (hexanes/DCM, 3/1→3/2) to obtain orange solids (130 mg, 0.22 mmol) in 64% yield. ^{1}H NMR (400 MHz, $CDCl_3$): 8.25 (1H, d, 7.7), 8.22 (1H, dd, 7.6, 1.1), 8.20 (1H, br dd, 7.7, 1.1), 8.128 (1H, d, 9.1), 8.124 (1H, d, 9.1), 8.06 (1H, br d, 8.8), 8.03 (1H, t, 7.6), 7.86 (2H, br m), 6.14 (1H, s), 3.44 (2H, t, 6.3), 3.09 (2H, br m), 2.61 (3H, s), 2.49 (3H, s), 2.40 (3H, s), 2.23 (3H, s), 2.06 (2H, kv, 7.0), 1.91 (2H, br m). ^{13}C{^{1}H} NMR (101 MHz, $CDCl_3$): 154.61, 153.53, 145.60, 140.63, 137.79, 132.41, 131.88, 131.32, 130.97, 130.26, 128.85, 128.81, 127.82, 127.65, 127.37, 126.12, 125.31, 125.20, 125.19, 124.93, 124.75, 124.66, 122.10, 33.09, 32.76, 30.26, 27.74, 16.60, 14.67, 14.62 (t, 2.5), 13.43 (t, 2.5), signals of two aromatic carbons are missing due to their broadness. HRMS-ESI: *exact mass* 582.16535 Da, found *m/z* 581.15790 $[M-H]^-$.

Probe **FP-1**

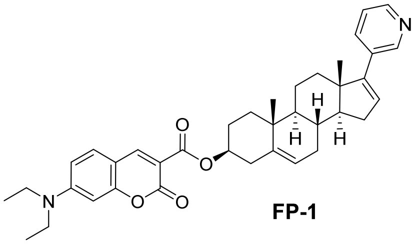

FP-1

To a solution of **P1** (100 mg, 0.29 mmol) and coumarine **F7** (90 mg, 0.34 mmol) in dry DMF (5 mL), DCC (75 mg, 0.36 mmol) and 4-DMAP (44 mg, 0.36 mmol,) were added. The mixture was stirred at RT for 2 h after which the temperature was heightened to 65 °C and stirring was continued O/N. The solvent was evaporated under reduced pressure and the pure product was obtained after two chromatographic purifications (AcOEt-chloroform, 1/3). Product **FP1** was obtained (91 mg, 0.15 mmol) as yellowish solids in 53% yield. R_F=0.7 in AcOEt. ^{1}H NMR (300 MHz, $CDCl_3$): 8.61 (1H, br d, 2.0), 8.44 (1H, br dd, 4.9, 1.5), 8.37 (1H, s), 7.66 (1H, ddd, 8.0, 2.2, 1.7), 7.34 (1H, d, 9.0), 7.23 (1H, ddd, 8.0, 4.9, 0.8), 6.58 (1H, dd, 9.0, 2.5), 6.44 (1H, d, 2.5), 5.99 (1H, dd, 3.3, 1.8), 5.43 (1H, br d, 4.9), 4.83 (1H, tdd, 11.2, 7.2, 4.4), 3.42 4H, q, 7.1), 2.26 (1H, ddd, 15.8, 6.5, 3.3), 1.21 (6H, t, 7.1), 1.10 (3H, s), 1.03 (3H, s), signals for next 16 protons in the area of 2.53 to 0.99 ppm were not resolved in the spectrum due to second order and overlapping of signals. ^{13}C{^{1}H} NMR (75 MHz, $CDCl_3$): 163.54, 158.51, 158.35, 152.89, 151.63, 148.94, 147.67, 147.60, 140.25, 134.08, 133.21, 131.07, 129.56, 123.24, 122.39, 109.55, 109.35, 107.74, 96.82, 86.73 (probably an imp.), 74.71, 57.57, 50.35, 49.14 (imp.), 47.44, 45.18, 38.32, 37.09, 36.95, 35.31, 34.06, 31.92, 31.64, 30.52, 27.92, 27.01 (imp.), 25.75 (imp.), 25.09, 20.93, 19.44, 16.68, 12.55, two extra signals in area 60-50 ppm belong to an impurity were not identified.

Probe **FP-2**

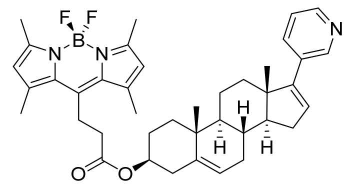

FP-2

A mixture of **P1** (100 mg, 0.29 mmol), BODIPY **F1**[1] (138 mg, 0.43 mmol, 1.5 eqviv.), HOBt (20 mg, 0.15 mmol), 4-DMAP (52 mg, 0.43 mmol) and EDCI (82 mg, 0.43 mmol) was stirred under argon atmosphere for 14 h (further as **method A**). The solvent was removed under reduced pressure and the residue was chromatographed (hexanes-AcOEt, 4/1). Material thus obtained was chromatographed once using the same mobile system. Product **FP-2** (117 mg, 0.18 mmol) was obtained in 62% yield. R_F = 0.2 in $CHCl_3$. ^{1}H NMR (500 MHz, $CDCl_3$): 8.62 (1H, br s, H21), 8.46 (1H, br d, 4.9, H22), 7.72 (1H, ddd, 7.9, 2.3, 1.5, H24), 7.28 (1H, dd, 7.9, 4.9, H23), 6.06 (2H, s, H31), 6.03 (1H, dd, 3.3, 1.7, H16), 5.42 (1H, dt, 5.4, 1.7, H6), 4.65 (1H, ddd, 11.4, 10.0, 6.1, 4.3, H3), 3.29 (2H, m, H27), 2.56 (2H, m, H26), 2.51 (6H, s, H33), 2.43 (6H, s, H34), 2.35 (1H, ddd, 13.4, 5.9, 1.4, H4), 2.32 (1H, m, H4'), 2.27 (1H, ddd, 15.8, 6.6, 3.3, H15), 2.07 (1H, m, H7), 2.07 (1H, m, H15'), 2.03 (1H, ddd, 12.2, 4.3, 2.7, H12), 1.91 (1H, m), 1.87 (1H, m, H2), 1.87 (1H, m, H1), 1.76 (1H, m, H8), 1.68 (1H, m), 1.68 (1H, m, H7'), 1.66 (1H, m, H11), 1.62 (1H, m, H11'), 1.60 (1H, m, H2'), 1.58 (1H, m, H14), 1.48 (1H, td, 12.2, 4.8, H12'), 1.16 (1H, m, H1'), 1.13 (1H, m), 1.09 (1H, m, H9), 1.08 (3H, s, H19), 1.04 (3H, s, H18). $^{13}C\{^1H\}$ NMR (126 MHz, $CDCl_3$): 171.24 (C25), 154.71 (t, 1.7, C32), 151.32 (C17), 146.85 (C21), 146.83 (C22), 143.59 (C28), 140.55 (C30), 139.91 (C5), 134.78 (C24), 133.55 (C20), 131.36 (C29), 130.12 (C16), 123.56 (C23), 122.59 (C6), 122.04 (C31), 74.71 (C3), , 57.55 (C14), 50.29 (C9), 47.44 (C13), 38.15 (C4), 36.95 (C1), 36.88 (C10), 35.92 (C26), 35.25 (C12), 31.95 (C15), 31.60 (C7), 30.47 (C8), 27.80 (C2), 23.84 (C27), 20.91 (C11), 19.36 (C19), 16.68 (C18), 16.52 (C34), 14.61 (t, 2.5, C33). $^{19}F\{^{11}B\}$ NMR (460 MHz, $CDCl_3$): an AB system with chemical exchange is observed as two broad peaks at -146.34 and -146.45 ppm. HRMS-ESI: *exact mass* 651.38076 Da, found *m/z* 652.38806 $[M+H]^+$, 674.36951 $[M+Na]^+$, 690.34332 $[M+K]^+$.

Probe **FP-3**

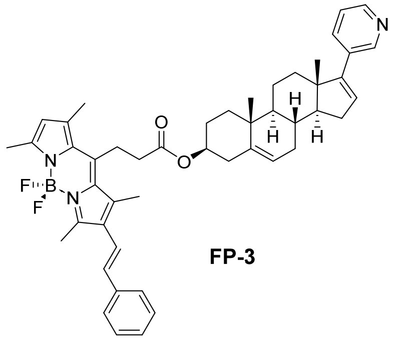

FP-3

Prepared by method A with 85 μmol of **P1** and 127 μmol of BODIPY **F4**. Chromatography in hexanes-AcOEt, 3/1. Product **FP-3** (30 mg, 40 μmol) was obtained as orange solids in 47% yield. R_F=0.4 in hexanes-AcOEt, 2/1. ^{1}H NMR (400 MHz, $CDCl_3$): 8.63 (1H, br s), 8.47 (1H, br d, 4.2), 7.71 (1H, dt, 8.0, 1.9), 7.48 (2H, ~d, 7.8), 7.37 (2H, ~t, 7.6), 7.29-7.25 (2H, an overlay of two signals and $CHCl_3$), 6.94 (1H, d, 16.5), 6.69 (1H, d, 16.5), 6.09 (1H, s), 6.03 (1H, dd, 3.3, 1.8), 5.43 (1H, br d, 5.3), 4.67 (1H, dddd, ΣJ = 32.2), 3.38 (2H, br m), 2.67 (3H, s), 2.61 (2H, ~dd, 10.2, 7.0), 2.54 (3H, s), 2.53 (3H, s), 2.47 (3H, s), 2.28 (1H, ddd, 15.8, 6.6, 3.3), 1.49 (2H, td, 12.0, 5.2), 1.09 (3H, s), 1.05 (3H, s), signals for next 15 protons in the area of 2.39 to 0.83 ppm were not resolved in the spectrum due to second order and overlapping of signals. $^{13}C\{^1H\}$ NMR (101 MHz, $CDCl_3$): 171.24, 155.01, 154.12, 151.44, 147.01, 143.51, 140.71, 139.94, 137.74, 136.14, 134.68, 133.53, 132.50, 131.77, 131.16, 130.03, 129.28, 128.86, 127.75, 126.32, 123.51, 122.64, 122.35, 119.85, 74.79, 57.60, 50.36, 47.49, 38.20, 37.01, 36.93, 36.01, 35.32, 31.98, 31.65, 30.53, 27.86, 24.03, 20.96, 19.40, 16.76, 16.72, 14.69 (br), 14.43, 14.10 (br). HRMS-ESI: *exact mass* 753.42771 Da, found *m/z* 754.4355 $[M+H]^+$, 776.41705 $[M+Na]^+$.

Probe **FP-4**

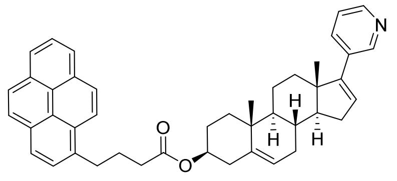

FP-4

Prepared by method A with 0.29 mmol of **P1** and 0.43 mmol of **F8**. Chromatography in hexanes-AcOEt, 3/1 → 1/1; (purified two times). Product **FP-4** (113 mg, 0.18 mmol) was isolated in 63% yield. R_F=0.6 in hexanes-AcOEt, 3/1. ^{1}H NMR (400 MHz, $CDCl_3$): 8.63 (1H, dd, 2.4, 0.9), 8.47 (1H, dd, 4.9, 1.7), 8.32 (1H, d, 9.3), 8.17 (1H, dd, 7.6, 1.2), 8.16 (1H, dd, 7.7, 1.2), 8.12 (1H, d, 9.3), 8.11 (1H, d, 7.8), 8.03 (1H, d, 9.0), 8.02 (1H, d, 9.0), 7.99 (1H, t, 7.6), 7.87 (1H, d, 7.8), 7.66 (1H, ddd, 7.9, 2.4, 1.7), 7.24 (1H, ddd, 7.9, 4.9, 0.9), 6.00 (1H, dd, 3.3, 1.8), 5.41 (1H, br d, 5.3), 4.67 (1H, dddd, 11.4, 9.8, 6.4, 4.5), 3.40 (2H, ~t, 7.7), 2.45 (2H, ~t, 7.7), 2.26 (1H, ddd, 15.8, 6.5, 3.3), 2.20 (1H, ~kv, 7.7), 1.47 (1H, td, 12.0, 5.1), 1.16 (1H, td, 13.8, 4.2), 1.07 (3H, s), 1.03 (3H, s), signals for next 14 protons in the area of 2.38 to 1.05 ppm were not resolved in the spectrum due to second order and overlapping of signals. ^{13}C{^{1}H} NMR (101 MHz, $CDCl_3$): 172.83, 151.47, 147.45, 147.38, 139.98, 135.80, 134.04, 133.13, 131.41, 130.89, 129.96, 129.49, 128.73, 127.47, 127.37, 127.35, 126.68, 125.82, 125.09, 124.99, 124.89, 124.80, 124.74, 123.34, 123.13, 122.27, 73.82, 57.42, 50.21, 47.31, 38.18, 36.90, 36.77, 35.17, 34.28, 32.80, 31.79, 31.47, 30.38, 27.79, 26.93, 20.79, 19.23, 16.54. HRMS-ESI: *exact mass* 619.34503 Da, found *m/z* 620.35232 $[M+H]^+$ and 642.33384 $[M+Na]^+$.

Probe **FP-5**

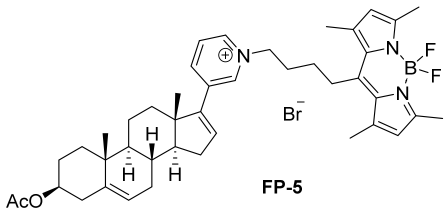

FP-5

Mixture of **P1** (80 mg, 0.23 mmol) and BODIPY **F2**[2] (80 mg, 0.27 mmol) in AcCN (8 mL) was stirred at 130°C for 24 h. The mixture was cooled to RT and diluted with DCM (5 mL). The product was poured into the mixture of ether/hexanes and stored at 4 °C for 20 minutes. The solids were filtered and this procedure was repeated twice. The solids were then dried *in vacuo,* redissolved in aqueous 1,4-dioxane and lyophilized (further as method B). Product **FP-5** (87 mg, 0.11 mmol) was obtained as brick solids in 56% yield. ^{1}H NMR (500 MHz, $CDCl_3$): 9.30 (2H, br s), 8.22 (1H, br d, 7.1), 7.77 (1H, br s), 6.69 (1H, br s), 6.01 (2H, s), 5.40 (1H, br d, 5.1), 5.11 (2H, br s), 4.60 (1H, tdd, 11.1, 5.8, 4.6), 3.01 (2H, br s), 2.47 (3H, s), 2.39 (3H, s), 2.35 (1H, m), 2.35 (1H, m), 2.32 (1H, m), 2.18 (2H, br s), 2.09 (1H, dd, 16.3, 11.8), 2.04 (1H, m), 2.03 (3H, s), 2.02 (1H, m), 1.88 (1H, m), 1.84 (1H, m), 1.74 (1H, m), 1.66 (1H, m), 1.65 (2H, br m), 1.64 (1H, m), 1.62 (1H, m), 1.59 (1H, m), 1.57 (1H, m), 1.43 (1H, m), 1.14 (1H, m), 1.06 (3H, s), 1.06 (1H, m), 1.04 (3H, s). ^{13}C{^{1}H} NMR (126 MHz, $CDCl_3$): 170.67, 154.29, 147.11, 145.06, 142.27 (CH), 142.17 (CH), 140.96, 140.82 (CH), 140.18, 138.27, 137.92 (CH), 131.49, 127.78 (CH), 122.13 (CH), 122.11 (CH), 73.88 (CH), 61.20 (CH_2), 57.39 (CH), 50.13 (CH), 47.55, 38.22 (CH_2), 37.02 (CH_2), 36.87, 34.95 (CH_2), 32.51 (CH_2), 32.36 (CH_2), 31.44 (CH_2), 30.34 (CH), 28.12 (CH_2), 27.84 (CH_2), 27.50 (CH_2), 21.54 (CH_3), 20.85 (CH_2), 19.36 (CH_3), 17.06 (CH_3), 16.63 (CH_3), 14.63 (CH_3). ^{19}F{^{11}B} NMR (460 MHz, $CDCl_3$): -146.27 (1F, d, $^{2}J_{FF}$ = 109 Hz, line width 40 Hz), -146.53 (1F, d, $^{2}J_{FF}$ = 109 Hz, line width 40 Hz). HRMS-ESI: *exact mass* ($C_{43}H_{55}BF_2N_3O_2^+$) 694.43499 Da, found *m/z* 694.43500 $[M]^+$.

Probe **FP-6**

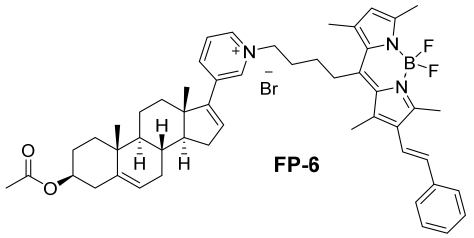

FP-6

Prepared by method B with 0.13 mmol of **P1** and 0.13 mmol of **F3**. Product **FP-6** (40 mg, 45 μmol) was obtained as deep red solids in 40% yield. R_F=0.4 in DCM/MeOH, 20/1. ^{1}H NMR (300 MHz, $CDCl_3$): 9.39 (1H, br s), 9.37 (1H, br d), 8.16 (1H, d, 8.1), 7.68 (1H, dd, 8.1, 5.1), 7.43 (2H, ~d, 7.7), 7.33 (2H, ~t, 7.4), 7.23 (1H, ~t, 7.0), 6.87 (1H, d, 16.6), 6.71 (1H, br m), 6.63 (1H, d, 16.6), 6.01 (1H, s), 5.36 (1H, br d, 4.8), 5.18 (2H, vbr), 4.57 (1H, m, Σ*J* = 32.4), 3.04 (2H, vbr), 2.61 (3H, s), 2.47 (3H, s), 2.44 (3H, s), 2.39 (3H, s), 2.03 (3H, s), 1.02 (3H, s), 0.99 (3H, s), signals for next 19 protons in the area of 2.38 to 1.05 ppm were not resolved in the spectrum due to their overlapping and second order features. ^{13}C{^{1}H} NMR (75 MHz, $CDCl_3$): 170.63, 154.55, 153.39, 146.99, 144.93, 142.23, 142.05, 141.21, 140.60, 140.07, 138.11, 137.85, 137.56, 136.43, 132.28, 131.82, 131.13, 128.97, 128.86, 127.77, 127.59, 126.29, 122.39, 122.10, 119.52, 77.16, 73.84, 60.83, 57.25, 50.01, 47.48, 38.17, 36.93, 36.80, 36.55, 34.89, 32.45, 31.38, 30.26, 28.00, 27.79, 27.62, 21.55, 20.80, 19.31, 17.20, 16.53, 14.87, 14.68 (br), 14.12 (br). HRMS-ESI: *exact mass* ($C_{51}H_{61}BF_2N_3O_2^+$) 796.48194 Da, found *m/z* 796.48273 $[M]^+$.

Probe **FP-7**

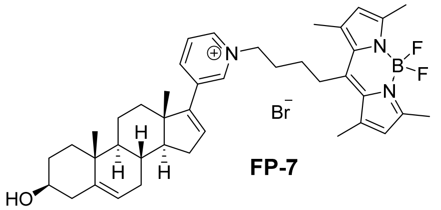

FP-7

Prepared by method B with 0.2 mmol of **P1** and 0.2 mmol of BODIPY **F2**. Product **FP-7** (71 mg, 97 μmol) was obtained as brick solids in 42% yield. ^{1}H NMR (500 MHz, CD_3SOCD_3): 9.02 (1H, br), 8.94 (1H, dt, 6.1, 1.2), 8.52 (1H, ddd, 8.3, 1.8, 1.2), 8.04 (1H, dd, 8.3, 6.1), 6.49 (1H, dd, 3.3, 1.8), 6.20 (2H, q, 0.8), 5.28 (1H, dt, 5.3, 1.8), 4.67 (2H, t, 7.3), 4.57 (0.3H, very broad), 3.23 (1H, tt, 11.1, 4.8), 2.94 (2H, m), 2.36 (6H, s), 2.32 (6H, s), 2.26 (1H, ddd, 16.5, 6.5, 3.4), 2.15 (1H, ddd, 13.3, 4.9, 2.1), 2.13-1.94 (6H, m), 1.74 (1H, dt, 13.3, 3.6), 1.67 (1H, td, 10.9, 4.9), 1.66-1.46 (7H, m), 1.38-1.27 (2H, m), 1.00-0.93 (2H, m), 1.00 (3H, s), 0.97 (3H, s). ^{13}C{^{1}H} NMR (126 MHz, CD_3SOCD_3): 153.29, 147.63, 145.67, 142.53, 141.73, 141.63, 141.54, 140.73, 136.21, 135.23, 130.64, 127.88, 121.75, 120.07, 69.95, 60.45, 57.01, 49.66, 46.71, 42.20, 36.79, 36.21, 33.66, 31.60, 31.37, 31.08, 30.82, 29.81, 27.81, 26.99, 20.26, 19.02, 15.89, 15.74, 14.06 (t, 2.0). ^{19}F{^{11}B} NMR (460 MHz, CD_3SOCD_3): an AB system with chemical exchange is observed as two broad peaks at -143.90 and -143.93 ppm; coalescence temperature is approx. 42 ºC. HRMS-ESI: *exact mass* ($C_{41}H_{53}BF_2N_3O^+$) 652.42443 Da, found *m/z* 652.42487 $[M]^+$.

Probe **FP-8**

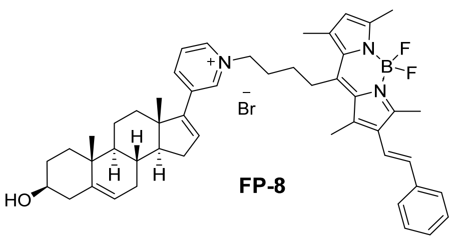

Prepared by method B with 0.23 mmol of **P1** and 0.27 mmol of BODIPY **F3**. Product **FP-8** (91 mg, 0.11 mmol) was obtained as red solids in 47% yield. ^{1}H NMR (400 MHz, $CDCl_3$): 9.34 (1H, br s), 9.29 (1H, br d, 6.0), 8.19 (1H, br d, 8.2), 7.72 (1H, dd, 8.2, 6.0), 7.45 (2H, ~d, 7.8), 7.33 (2H, ~t, 7.6), 7.24 (1H, ~tt, 7.3, 2.1), 6.88 (1H, d, 16.5), 6.73 (1H, dd, 3.1, 1.8), 6.66 (1H, d, 16.5), 6.03 (1H, s), 5.34 (1H, m, ΣJ = 10.7), 5.15 (2H, m, ΣJ = 48.1), 3.50 (1H, m, ΣJ = 31.4), 3.07 (2H, br m), 2.62 (3H, s), 2.49 (3H, s), 2.47 (3H, s), 2.42 (3H, s), 1.022 (3H, s), 1.017 (3H, s), signals for next 22 protons in the area of 2.54 to 0.97 ppm were not resolved in the spectrum due to their overlapping and second order features. ^{13}C{^{1}H} NMR (101 MHz, $CDCl_3$): 154.62, 153.49, 146.98, 144.88, 142.13, 141.84, 141.22, 140.74, 138.28, 138.09, 137.60, 136.45, 132.40, 131.87, 131.20, 129.09, 128.87, 127.78, 127.65, 126.33, 122.44, 121.15, 119.54, 71.69, 60.94, 57.37, 50.15, 47.55, 42.36, 37.24, 36.74, 34.98, 32.50, 32.28, 31.69, 31.41, 30.34, 28.04, 27.62, 20.88, 19.43, 17.38, 16.64, 15.07, 14.70, 14.12, a signal of one aromatic carbon is covered within area 142.0 to 140.5 ppm. HRMS-ESI: *exact mass* ($C_{49}H_{59}BF_2N_3O^+$) 754.47138 Da, found *m/z* 754.47234 $[M]^+$.

Probe **FP-9**

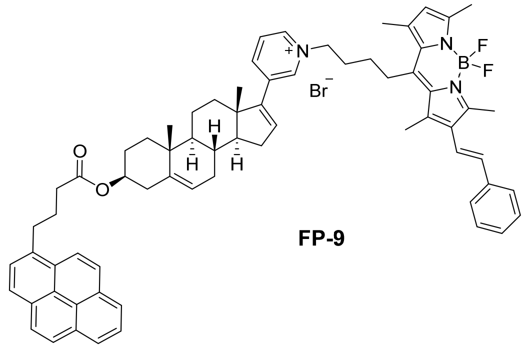

Prepared by method B with 0.13 mmol of **FP-4** and 0.13 mmol of BODIPY **F3**. Product **FP-9** (90 mg, 81 μmol) was obtained as red solids in 63% yield. ^{1}H NMR (400 MHz, $CDCl_3$): 9.26 (2H, br s), 9.30 (1H, d, 9.3), 8.15 (1H, dd, 7.6, 1.0), 8.14 (1H, dd, 7.7, 1.1), 8.11 (1H, s), 8.09 (1H, d, 1.2), 8.01 (1H, d, 9.0), 8.00 (1H, d, 9.0), 7.97 (1H, t, 7.6), 7.85 (1H, d, 7.8), 7.70 (1H, br s), 7.44 (2H, ~d, 7.8), 7.32 (2H, ~t, 7.6), 7.23 (1H, ~t, 7.3), 6.86 (1H, d, 16.5), 6.67 (1H, br s), 6.64 (1H, d, 16.5), 6.01 (1H, s), 5.35 (1H, br d, 4.1), 5.10 (2H, br s), 4.63 (1H, m, ΣJ = 32.1), 3.38 (2H, ~t, 7.7), 3.02 (2H, br s), 2.61 (3H, s), 2.48 (3H, s), 2.43 (3H, s), 2.38 (3H, s), 1.01 (3H, s), 0.98 (3H, s), signals for next 26 protons in the area of 2.61 to 0.95 ppm were not resolved in the spectrum due to their overlapping and second order features. ^{13}C{^{1}H} NMR (101 MHz, $CDCl_3$): 172.92, 154.52, 153.35, 146.95, 144.90, 141.97, 141.94, 141.22, 140.75, 140.03, 138.06, 137.77, 137.55, 136.46, 135.90, 132.27, 131.81, 131.50, 131.12, 130.98, 130.05, 128.95, 128.83, 128.82, 127.74, 127.57, 127.48, 127.45, 126.80, 126.30, 125.94, 125.17, 125.07, 125.01, 124.91, 124.85, 123.44, 122.39, 122.09, 119.49, 73.80, 60.91, 57.21, 49.97, 47.46, 38.21, 36.91, 36.77, 34.87, 34.37, 32.89, 32.41, 32.33, 31.34, 30.22, 27.99, 27.84, 27.63, 27.03, 20.76, 19.28, 17.39, 16.55, 15.09, 14.66, 14.10, a signal of one aromatic carbon was not recognized due to an overlap. HRMS-ESI: *exact mass* ($C_{69}H_{73}BF_2N_3O_2^+$) 1024.57584 Da, found *m/z* 1024.57721 $[M]^+$.

Probe **FP-10**

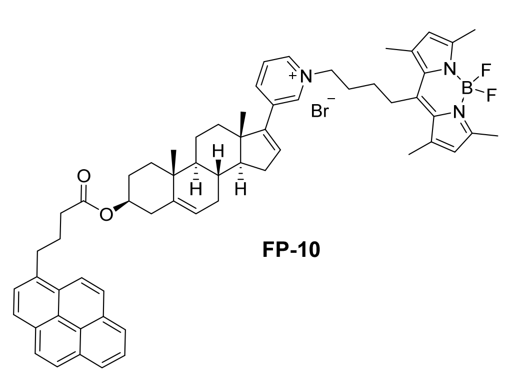

FP-10

Prepared by method B with 0.13 mmol of **FP-4** and 0.13 mmol of BODIPY **F2**. Product **FP-10** (77 mg, 76 μmol) was obtained as dark solids in 59% yield. ^{1}H NMR (400 MHz, CD_2Cl_2): 9.42 (1H, br s), 9.28 (1H, br d, 5.2), 8.35 (1H, dd, 9.3, 1.3), 8.24 (1H, br d, 8.0), 8.20 (1H, overlaped), 8.19 (1H, overlaped), 8.14 (2H, overlaped), 8.06 (1H, overlaped), 8.04 (1H, overlaped), 8.01 (1H, overlaped), 7.90 (1H, dd, 7.8, 1.4), 7.82 (1H, br ~t, 6.8), 6.72 (1H, br s), 6.06 (2H, s), 5.42 (1H, br d, 5.2), 5.11 (2H, br m), 4.63 (1H, m), 3.40 (2H, ~t, 7.8), 3.04 (2H, br m), 2.46 (3H, s), 2.42 (3H, s), 1.45 (1H, td, 11.6, 4.5), 1.08 (3H, s), 1.07 (3H, s), signals for next 25 protons in the area of 2.48 to 1.05 ppm were not resolved in the spectrum due to their overlapping and second order features. ^{13}C{^{1}H} NMR (101 MHz, CD_2Cl_2): 173.15, 154.56, 147.78, 145.76, 142.63, 142.39, 141.52, 141.32, 140.72, 138.57, 137.94, 136.72, 131.95, 131.86, 131.45, 130.46, 129.26, 128.12, 128.00, 127.76, 127.15, 126.43, 125.52, 125.42, 125.37, 125.27, 123.98, 122.48, 122.32, 74.24, 61.35, 57.84, 50.59, 47.92, 38.69, 37.44, 37.28, 35.30, 34.73, 33.26, 32.80, 32.74, 31.84, 30.74, 28.46, 28.31, 28.04, 27.52, 21.25, 19.58, 17.16, 16.76, 14.79, signals of two aromatic carbons were not recognized due to an overlap. HRMS-ESI: *exact mass* ($C_{61}H_{67}BF_2N_3O_2^+$) 922.52889 Da, found *m/z* 922.52726 $[M]^+$.

Probe **FP-11**

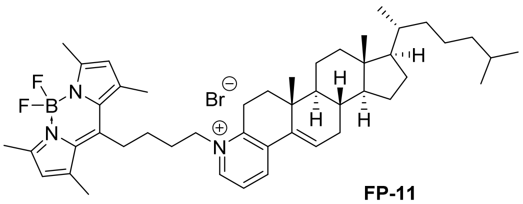

FP-11

Prepared by method B with 0.21 mmol of **P2** and 0.31 mmol of BODIPY **F2**. Product **FP-11** (110 mg, 0.14 mmol) was obtained as brick solids in 67% yield. ^{1}H NMR (500 MHz, $CDCl_3$): 9.70 (1H, br s), 8.35 (1H, br d, 7.7), 7.68 (1H, br m), 6.38 (1H, br m), 5.99 (2H, s), 5.04 (1H, br s), 4.88 (1H, br s), 3.26 (1H, br d, 16.7), 3.08 (1H, m), 3.01 (2H, br m), 2.44 (3H, s), 2.40 (3H, s), 2.30 (1H, m), 2.25 (1H, m), 2.06 (2H, m), 2.05 (1H, m), 1.85 (1H, m), 1.81 (1H, m), 1.75 (2H, m), 1.61 (1H, m), 1.58 (1H, m), 1.53 (1H, m), 1.50 (1H, m), 1.48 (1H, m), 1.46 (1H, m), 1.37 (1H, m), 1.33 (1H, m), 1.33 (1H, m), 1.28 (1H, m), 1.19 (1H, m), 1.14 (1H, m), 1.11 (2H, m), 1.11 (1H, m), 1.08 (1H, m), 1.04 (1H, m), 1.02 (1H, m), 0.99 (1H, m), 0.91 (3H, d, 6.5), 0.85 (3H, s), 0.84 (3H, d, 6.6), 0.84 (3H, d, 6.6), 0.70 (3H, s). ^{13}C{^{1}H} NMR (126 MHz, $CDCl_3$): 154.12, 150.22, 145.11, 144.96 (CH), 140.99, 140.74 (CH), 137.08, 136.28, 131.55, 129.55 (CH), 125.44 (CH), 122.03 (CH), 56.95 (CH_2), 56.50 (CH), 56.17 (CH), 48.64 (CH), 42.47, 39.58 (CH_2), 39.53 (CH_2), 36.24 (CH_2), 35.83 (CH), 34.57, 33.02 (CH_2), 32.42 (CH_2), 31.10 (CH), 30.69 (CH_2), 28.27 (CH_2), 28.09 (CH_2), 28.09 (CH), 27.55 (CH_2), 24.38 (CH_2), 24.27 (CH_2), 23.91 (CH_2), 22.89 (CH_3), 22.64 (CH_3), 21.19 (CH_2), 18.79 (CH_3), 18.54 (CH_3), 17.07 (CH_3), 14.55 (CH_3), 11.99 (CH_3). ^{19}F{^{11}B} NMR (460 MHz, $CDCl_3$): -145.40 (1F, d, $^2J_{FF}$ = 107 Hz, line width 60 Hz), -146.67 (1F, d, $^2J_{FF}$ = 107 Hz, line width 60 Hz); coalescence temperature is above 60 °C. HRMS-ESI: exact mass for $C_{47}H_{67}BF_2N_3^+$ 722.53906, found *m/z* 722.53782 $[M]^+$.

Probe **FP-12**

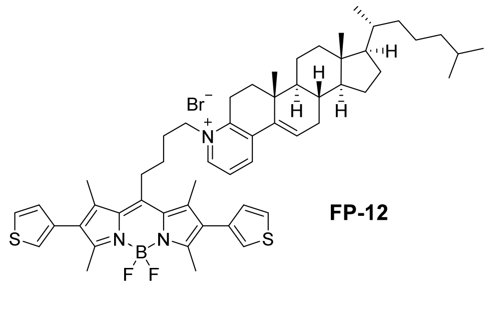

FP-12

Prepared by method B with 0.24 mmol of **P2** and 0.24 mmol of BODIPY **F5.** Product **FP-12** (46 mg, 48 μmol) was obtained as pink solids in 47% yield. ^{1}H NMR (400 MHz, CD_2Cl_2): 9.84 (1H, dd, 6.1, 1.0), 8.41 (1H, dd, 8.4, 1.0), 7.73 (1H, dd, 8.3, 6.1), 7.46 (2H, dd, 4.9, 3.0), 7.23 (2H, dd, 3.0, 1.3), 7.08 (2H, dd, 4.9, 1.3), 6.42 (1H, dd, 5.4, 2.7), 5.16 (2H, br m), 4.93 (2H, br m), 2.48 (6H, s), 2.45 (6H, s), 0.96 (3H, d, 6.5), 0.89 (3H, d, 6.7), 0.89 (3H, s), 0.88 (3H, d, 6.7), 0.74 (3H, s), signals for next 30 protons in the areas of 3.31 to 3.07 ppm and 2.50 to 0.84 ppm were not resolved in the spectrum due to their overlapping, second order features, and impurities. ^{13}C{^{1}H} NMR (101 MHz, CD_2Cl_2): 153.62 (br), 150.59, 146.08, 145.36, 141.24, 137.93 (br), 137.68, 136.67, 134.12, 131.95, 130.18, 129.91, 129.48, 126.04, 125.71, 124.85, 57.16, 57.00, 56.67, 49.10, 42.92, 40.04, 36.72, 36.34, 35.04, 33.47, 32.88, 31.57, 31.19, 28.75, 28.73, 28.59, 28.42, 24.83, 24.69, 24.37, 23.11, 22.86, 21.66, 19.05, 18.70, 15.55, 13.76 (t, 2.5), 12.21, a signal of one aliphatic carbon was not recognized due to an overlap or low intensity. HRMS-ESI: exact mass for $C_{55}H_{71}BF_2N_3S_2^+$ 886.51450 Da, found *m/z* 886.51575 [M]$^+$.

Probe **FP-13**

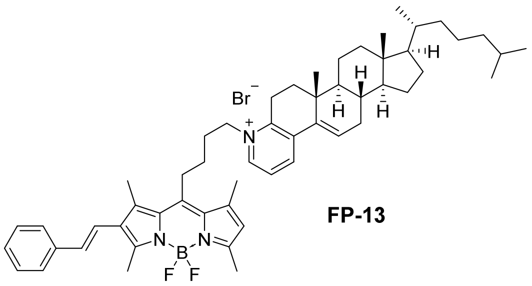

FP-13

Prepared by method B with 0.12 mmol of **P2** and 0.13 mmol of BODIPY **F3**. Product **FP-13** (43 mg, 48 μmol) was obtained as pink solids in 40% yield. ^{1}H NMR (400 MHz, CD_2Cl_2): 9.82 (1H, br d, 5.6), 8.40 (1H, d, 8.0), 7.75 (1H, dd, 8.0, 5.6), 7.51 (2H, ~d, 7.6), 7.37 (2H, ~t, 7.6), 7.27 (1H, ~t, 7.3), 6.98 (1H, d, 16.6), 6.73 (1H, d, 16.6), 6.45 (1H, dd, 5.1, 2.5), 6.12 (1H, s), 5.09 (2H, br m), 4.98 (2H, br m), 2.63 (3H, s), 2.55 (3H, s), 2.49 (6H, s), 2.49 (3H, s), 0.95 (3H, d, 6.5), 0.89 (3H, d, 6.6), 0.88 (3H, d, 6.7), 0.73 (3H, s), signals for next 30 protons in the areas of 3.27 to 3.02 ppm and 2.55 to 0.85 ppm were not resolved in the spectrum due to their overlapping, second order features, and impurities. ^{13}C{^{1}H} NMR (101 MHz, CD_2Cl_2): 155.01, 153.82, 150.52, 145.64, 145.41, 141.24, 138.23, 137.67, 137.01, 136.63, 132.54, 132.42, 131.71, 130.19, 129.40, 129.22, 128.09, 126.70, 125.74, 122.71, 120.14, 57.16, 56.97, 56.65, 49.10, 42.90, 40.04, 36.71, 36.33, 35.01, 33.46, 32.87, 31.55, 31.02, 28.72, 28.61, 28.59, 28.20, 24.77, 24.68, 24.36, 23.11, 22.86, 21.68, 19.04, 18.69, 17.50, 15.27, 14.85, 14.35, 12.20, signals of one aliphatic and one aromatic carbon were not recognized due to an overlap or low intensity. HRMS-ESI: exact mass for $C_{55}H_{73}BF_2N_3^+$ 824.58601 Da, found *m/z* 824.58696 [M]$^+$.

Probe **FP-14**

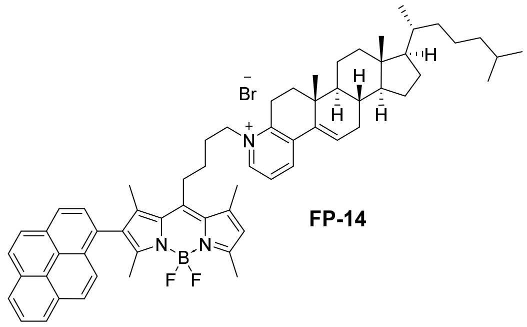

FP-14

Prepared by method B with 0.1 mmol of **P2** and 0.13 mmol of BODIPY **F6**. Product **FP-13** (36 mg, 36 μmol) was obtained as pink solids in 36% yield. ^{1}H NMR (500 MHz, CD_3SOCD_3, 90°C): Most of signals at 27 °C are very broad. Increasing temperature to about 90 °C leads to narrowing of the signals, however, two sets of signals (A, and broader B) close 1:1 ratio are observed. Unfortunately, **FP-14** is unstable at 90°C, which hinders a more detailed analysis. All is demonstrated by the attached spectra (*vide infra*). 8.86 (1H^{A+B}, d, 6.0), 8.65 (1H^{B}, d, 8.2), 8.60 (1H^{A}, d, 8.2), 8.34 (1H^{A+B}, d, 7.7), 8.31 (1H^{A+B}, d, 7.7), 8.28 (1H^{A}, d, 7.6), 8.27 (1H^{B}, d, 7.4), 8.21 (2H^{A}, strong AB system), 8.20 (2H^{B}, two strong AB), 8.14 (1H^{A}, d, 9.3), 8.11 (1H^{B}, d, 9.2), 8.073 (2H^{A}, t, 7.6), 8.067 (2H^{B}, t, 7.6), 7.88 (2H^{A}, d, 7.8), 7.87 (2H^{B}, d, 7.8), 7.84 (1H^{B}, dd, 8.2, 6.0), 7.83 (1H^{A}, dd, 8.4, 5.3), 7.70 (1H^{A}, d, 9.2), 7.66 (1H^{B}, d, 9.2), 6.49 (1H^{B}, dd, 5.3, 2.6), 6.44 (1H^{A}, br dd), 6.30 (1H^{A+B}, s), 4.70-4.58 (2H^{A+B}, m), 2.21 (6H^{A+B}, br s), 2.15 (3H^{A+B}, s), 2.12 (3H^{A+B}, s), 0.89 (3H$^{A/B}$, d, 6.3), 0.88 (3H$^{A/B}$, d, 6.3), 0.84 (four overlaid 3H^{A+B}, d), other signals are overlaid within an area 3.30-0.55 ppm. ^{13}C{^{1}H} NMR (126 MHz, CD_3SOCD_3): Low solubility, mostly broad signals, presence of two isomers, and instability of **FP-14** impaired recording and interpretation of the ^{13}C spectrum. HRMS-ESI: exact mass for $C_{63}H_{75}BF_2N_3^+$ 922.60166 Da, found *m/z* 922.60309[M]$^+$.

1.2 Compounds characterization

Various 1D and 2D NMR spectra (e.g., ^{1}H, ^{19}F and ^{13}C, COSY, HSQC, HMBC, ROESY) were recorded on a Varian Gemini 300 (Varian, Palo Alto, USA), Agilent 400-MR DDR2 (Agilent, Santa Clara, USA) and JEOL ECZ 500R (JEOL, Tokyo, Japan) to confirm and characterize the molecular structure of the prepared compounds. Chemical shifts (δ) are given in ppm and interaction constants (*J*) in Hz. High-resolution mass spectra (HRMS) were measured by LTQ ORBITRAP VELOS with HESI$^+$/HESI$^-$ ionization (Thermo Scientific, Waltham, USA). For thin-layer chromatograms were used plates coated with silica gel by Stahl (10-40 μm, Merck) and aluminum TLC sheets-coated silica gel bounded with starch for detection in UV light (Silufol UV 254 nm, Merck). For visualization, 50% sulfuric acid in MeOH was used and plates were successively heated. For column chromatography, silica gel (32-62 μm, SiliTech, MP Biomedicals) was used. Solvents were purchased from PENTA, steroids from Steraloids (Newport, USA) and other reagents from Sigma-Aldrich (Corp., St. Louis, USA).

NMR and HMRS spectra are available upon request.

Table S1. Calculated lipophilicity of synthetic probes

Probe	Mol. Weight	LogP GALAS	LogP Consensus
FP-1	592.77	7.06	8.32
FP-2	651.64	6.30	6.30
FP-3	753.77	7.86	7.86
FP-4	619.83	8.99	8.99
FP-5	694.72	3.02	3.02
FP-6	782.83	4.35	4.35
FP-7	652.69	2.87	2.87
FP-8	754.82	3.94	3.94

FP-9	1025.14	8.36	8.36
FP-10	923.01	6.97	6.97
FP-11	722.86	4.48	4.48
FP-12	887.11	6.55	6.55
FP-13	825.00	5.87	5.87
FP-14	923.10	7.99	7.99
TF-Chol	576.61	6.31	6.31

Software name and version: ACD/Percepta 14.1.0 (Build 2921); for explanation see http://perceptahelp.acdlabs.com/help_v2016/index.php/Main_Page (Jan 17, 2018).

1. Crystallography

X-ray analysis. A selected single crystal was measured at 120K with a four-circle diffractometer Gemini of Oxford Diffraction, equipped with a sealed copper X-ray tube, a mirror collimator Cu-Ultra, and a CCD detector Atlas S1. The sample quality was mediocre with some spurious diffraction peaks, nevertheless fully sufficient for structure solution using Superflip[3] and structure refinement with Jana2006[4]. All non-hydrogen atoms were refined with harmonic atomic displacement parameters (ADP). Hydrogen atoms bonded to carbon were adjusted to geometrically expected positions with U_{iso} equal to the 1.2 multiple of U_{eq} of the corresponding parent atom. The hydrogen atoms attached to oxygen were confirmed by a difference Fourier maps and refined using the above-mentioned constraint for ADP and a distance restraint 0.86 Å for the O-H bond length. Since the structure was non-centrosymmetric, space group $P2_1$, we also refined the Flack parameter based on 3367 Friedel pairs, which resulted 0.07(16) confirming the correct absolute configuration of the structure model. The large e.s.d. of the Flack parameter was caused by the presence of only light atoms in the crystal structure. The crystallographic parameters are summarized in Table S2. Structure plots were done with Diamond 4 of Crystal Impact[5].

The crystallographic data were deposited at Cambridge Crystallographic Data Centre http://www.ccdc.cam.ac.uk/ under the following deposition number CCDC 1580089.

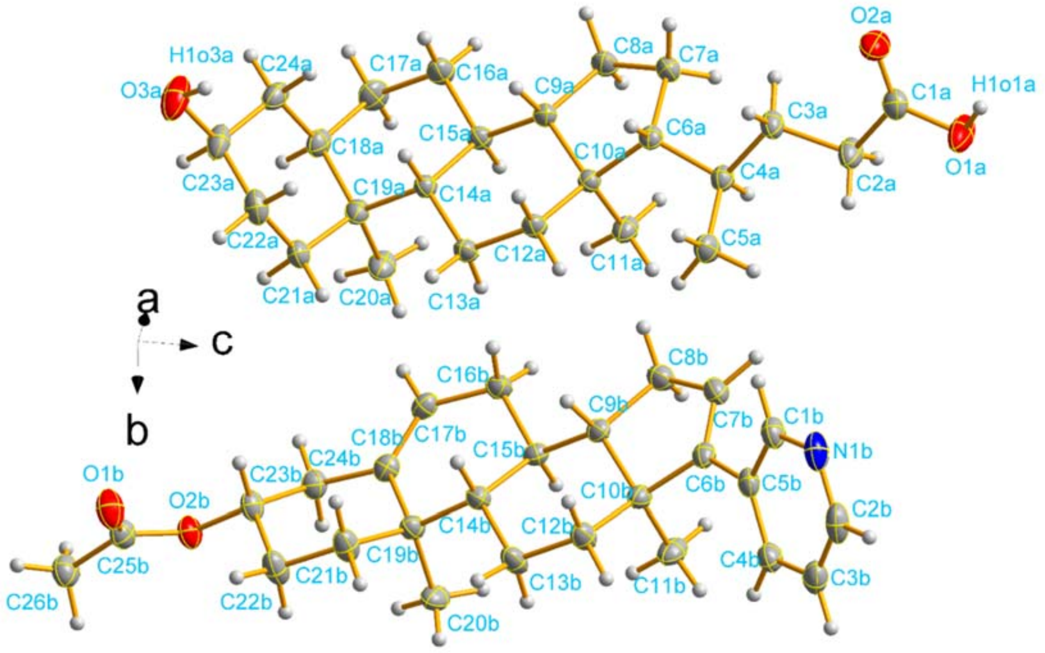

Fig. S4. Two molecules of the cocrystal with atom labels: the lithocholic acid and P1 are distinguished by the suffix "a" and "b", respectively. The common rings of both molecules have the same labelling. Non-hydrogen atoms are displayed as ADP ellipsoids drawn at the 50% probability level. Hydrogen atoms are labelled only when participating in hydrogen bonds. All atoms are located in general positions.

The structure features two intermolecular moderately strong hydrogen bonds, O1a-H1O1a…N1b [D-H 0.86(1) Å, H…A 1.871(10) Å, D-H…A 165(3) °] and O3a-H1O3a…O1b [D-H 0.861(18) Å, H…A 1.020(16) Å, D-H…A 165(2) °]. These bonds connect the molecules of the lithocholic acid and P1 into chains extended along the diagonal of the ac plane (Fig. S5A). Only Van der Waals interaction acts between the chains: the complete packing is quite compact (Fig. S5B), it is composed of the above-mentioned separate chains.

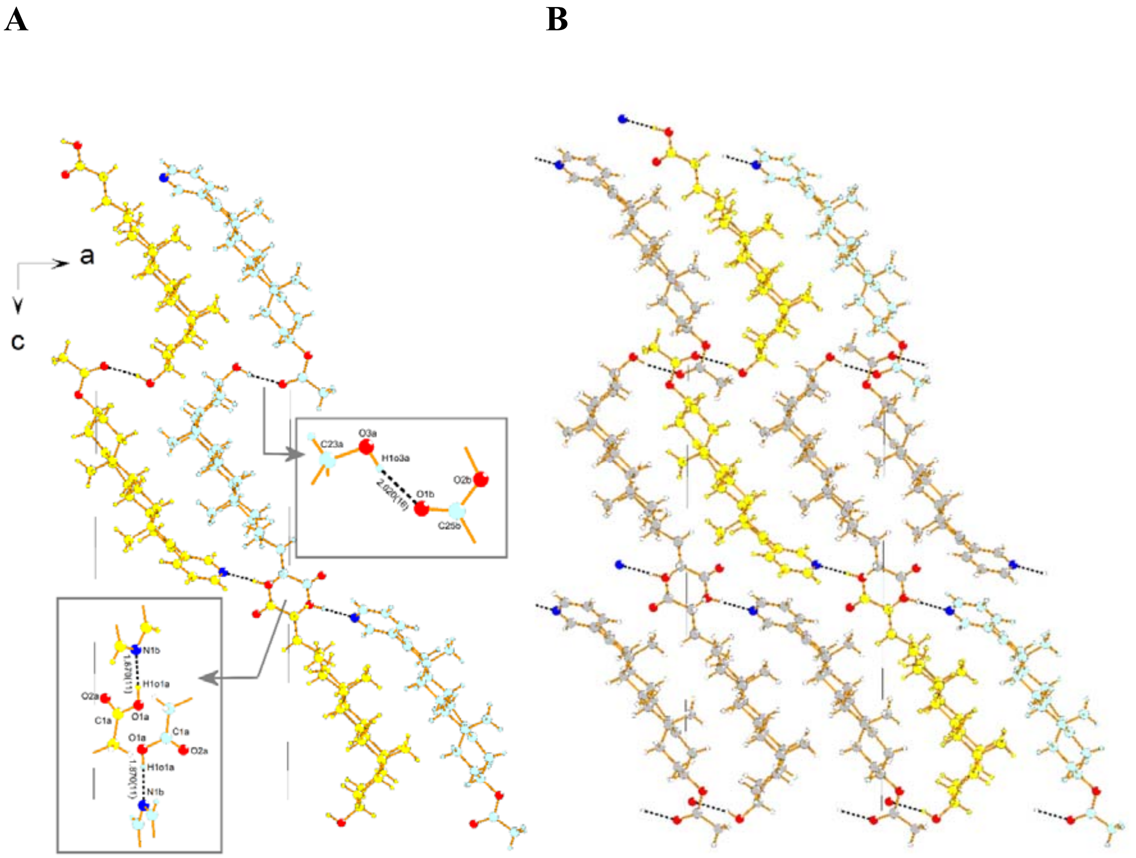

Fig. S5. Packing of the cocrystal viewed along *b*. Color codes: nitrogen blue; oxygen red; hydrogen white/light blue/yellow; carbon white/light blue/yellow; hydrogen bonds black dashed lines. (A) Two neighboring chains (yellow and light blue) of molecules. Insets show that hydrogen bonds connect molecules into chains but do not act between the neighbouring chains; (B) the complete packing caused by hydrogen bonds and Van der Waals interactions.

Table S2. **The crystallographic details**

**Crystal data**	
Chemical formula	$C_{50}H_{73}NO_5$
M_r	768.1
Crystal system, space group	Monoclinic, $P2_1$
Temperature (K)	293
a, *b*, *c* (Å)	11.1329 (2), 7.5633 (2), 25.2927 (5)
β (°)	90.401 (2)
V (Å^3)	2129.63 (8)
Z	2
Radiation type	Cu *K*α
μ (mm^{-1})	0.59
Crystal size (mm)	0.41 × 0.25 × 0.12
**Data collection**	
Diffractometer	Xcalibur, Atlas, Gemini ultra diffractometer

Absorption correction	Multi-scan by *CrysAlis PRO* 1.171.39.33b (Rigaku Oxford Diffraction, 2017)
T_{min}, T_{max}	0.661, 1
No. of measured, independent and observed [$I > 3\sigma(I)$] reflections	30130, 7437, 6947
R_{int}	0.064
$(\sin \theta/\lambda)_{max}$ (Å^{-1})	0.597
**Refinement**	
$R[F^2 > 2\sigma(F^2)]$, $wR(F^2)$, S	0.037, 0.089, 1.60
No. of reflections	7437
No. of parameters	513
No. of restraints	2
H-atom treatment	H atoms treated by a mixture of independent and constrained refinement
$\Delta\rho_{max}$, $\Delta\rho_{min}$ (e Å^{-3})	0.19, −0.18
Absolute structure	3367 of Friedel pairs used in the refinement
Absolute structure parameter	0.07 (16)

Table S3. **The distances and angles (without hydrogen atoms on carbon)**

O2b-C25b	1.340(2)
O2b-C23b	1.460(2)
O1b-C25b	1.209(2)
O2a-C1a	1.203(2)
O1a-C1a	1.327(2)
O1a-H1o1a	0.860(10)
O3a-C23a	1.425(2)
O3a-H1o3a	0.861(18)
N1b-C2b	1.338(3)
N1b-C1b	1.336(2)
C19a-C14a	1.561(2)
C19a-C21a	1.535(2)
C19a-C18a	1.547(3)
C19a-C20a	1.539(2)
C10a-C6a	1.563(2)
C10a-C12a	1.531(2)
C10a-C9a	1.542(2)
C10a-C11a	1.540(2)
C25b-C26b	1.496(3)
C4a-C6a	1.540(2)
C4a-C3a	1.538(2)
C4a-C5a	1.527(2)
C4a-H1c4a	0.96
C1a-C2a	1.504(3)
C15b-C14b	1.538(2)
C15b-C9b	1.522(2)
C15b-C16b	1.532(2)
C6a-C7a	1.562(2)
C14a-C15a	1.545(2)
C14a-C13a	1.535(2)

C5b-C4b	1.392(3)
C5b-C6b	1.474(2)
C5b-C1b	1.395(3)
C19b-C14b	1.555(2)
C19b-C18b	1.530(2)
C19b-C21b	1.539(2)
C19b-C20b	1.541(3)
C4b-C3b	1.389(3)
C15a-C9a	1.525(2)
C15a-C16a	1.523(2)
C24b-C18b	1.516(2)
C24b-C23b	1.519(2)
C10b-C6b	1.531(2)
C10b-C9b	1.542(2)
C10b-C12b	1.524(2)
C10b-C11b	1.547(3)
C6b-C7b	1.339(3)
C14b-C13b	1.546(2)
C13b-C12b	1.544(3)
C18b-C17b	1.326(2)
C9b-C8b	1.531(2)
C2b-C3b	1.378(3)
C7b-C8b	1.507(3)
C23b-C22b	1.507(3)
C22b-C21b	1.525(3)
C12a-C13a	1.538(2)
C3a-C2a	1.518(3)
C24a-C18a	1.535(3)
C24a-C23a	1.518(3)
C9a-C8a	1.522(2)
C21a-C22a	1.522(3)
C17b-C16b	1.499(2)
C18a-C17a	1.532(3)
C23a-C22a	1.516(3)
C16a-C17a	1.530(3)
C7a-C8a	1.543(3)
C7a-H1c7a	0.96
C7a-H2c7a	0.96
C25b-O2b-C23b	117.01(14)
C1a-O1a-H1o1a	106.9(17)
C23a-O3a-H1o3a	105.7(15)
C2b-N1b-C1b	117.88(17)
C14a-C19a-C21a	112.62(14)
C14a-C19a-C18a	109.57(14)
C14a-C19a-C20a	110.58(14)
C21a-C19a-C18a	108.07(14)
C21a-C19a-C20a	106.20(14)
C18a-C19a-C20a	109.71(15)
C6a-C10a-C12a	116.02(13)
C6a-C10a-C9a	100.31(13)
C6a-C10a-C11a	110.12(13)
C12a-C10a-C9a	106.68(13)
C12a-C10a-C11a	111.19(14)

C9a-C10a-C11a	112.01(14)
O2b-C25b-O1b	124.08(17)
O2b-C25b-C26b	111.66(15)
O1b-C25b-C26b	124.25(17)
C6a-C4a-C3a	109.38(14)
C6a-C4a-C5a	112.79(14)
C3a-C4a-C5a	110.08(14)
O2a-C1a-O1a	122.93(17)
O2a-C1a-C2a	125.01(16)
O1a-C1a-C2a	112.07(16)
C14b-C15b-C9b	107.75(13)
C14b-C15b-C16b	110.20(13)
C9b-C15b-C16b	111.72(14)
C10a-C6a-C4a	119.47(14)
C10a-C6a-C7a	103.66(13)
C4a-C6a-C7a	111.56(13)
C19a-C14a-C15a	111.86(14)
C19a-C14a-C13a	113.62(14)
C15a-C14a-C13a	111.26(13)
C4b-C5b-C6b	123.79(16)
C4b-C5b-C1b	116.76(17)
C6b-C5b-C1b	119.44(16)
C14b-C19b-C18b	110.74(14)
C14b-C19b-C21b	108.80(14)
C14b-C19b-C20b	110.98(14)
C18b-C19b-C21b	107.57(14)
C18b-C19b-C20b	108.44(14)
C21b-C19b-C20b	110.25(14)
C5b-C4b-C3b	119.54(18)
C14a-C15a-C9a	108.89(13)
C14a-C15a-C16a	111.19(14)
C9a-C15a-C16a	112.25(14)
C18b-C24b-C23b	111.74(15)
C6b-C10b-C9b	99.68(13)
C6b-C10b-C12b	117.24(15)
C6b-C10b-C11b	108.20(14)
C9b-C10b-C12b	106.65(14)
C9b-C10b-C11b	112.69(14)
C12b-C10b-C11b	111.86(15)
C5b-C6b-C10b	125.93(15)
C5b-C6b-C7b	124.64(16)
C10b-C6b-C7b	109.36(15)
C15b-C14b-C19b	112.66(14)
C15b-C14b-C13b	112.56(14)
C19b-C14b-C13b	112.17(14)
C14b-C13b-C12b	114.87(15)
C19b-C18b-C24b	115.78(14)
C19b-C18b-C17b	123.11(15)
C24b-C18b-C17b	121.10(16)
C15b-C9b-C10b	113.01(14)
C15b-C9b-C8b	122.40(14)
C10b-C9b-C8b	104.33(13)
N1b-C2b-C3b	122.65(18)

C6b-C7b-C8b	112.57(16)
O2b-C23b-C24b	106.90(14)
O2b-C23b-C22b	108.68(14)
C24b-C23b-C22b	111.74(15)
C23b-C22b-C21b	110.10(15)
C10a-C12a-C13a	111.93(14)
C14a-C13a-C12a	113.86(14)
C4a-C3a-C2a	114.64(15)
C18a-C24a-C23a	112.32(16)
C10a-C9a-C15a	114.38(13)
C10a-C9a-C8a	104.02(13)
C15a-C9a-C8a	118.92(14)
C19a-C21a-C22a	115.35(15)
C10b-C12b-C13b	110.24(15)
C9b-C8b-C7b	99.93(14)
C1a-C2a-C3a	113.69(15)
C18b-C17b-C16b	125.06(16)
C4b-C3b-C2b	119.02(19)
N1b-C1b-C5b	124.14(18)
C19a-C18a-C24a	113.16(15)
C19a-C18a-C17a	111.99(14)
C24a-C18a-C17a	111.63(15)
O3a-C23a-C24a	112.49(17)
O3a-C23a-C22a	112.52(16)
C24a-C23a-C22a	110.04(15)
C19b-C21b-C22b	113.24(16)
C21a-C22a-C23a	110.43(16)
C15a-C16a-C17a	111.61(15)
C6a-C7a-C8a	107.07(14)
C15b-C16b-C17b	112.44(14)
C9a-C8a-C7a	103.55(14)
C18a-C17a-C16a	111.89(15)

2. Spectral analysis

UV-VIS Spectrometry. The stability constants of the probe with cholesterol and cholesterol acetate were studied by UV-Vis spectroscopy in aqueous medium (H_2O:DMSO; 90:10 (v/v)) and MeOH and PBS (5:95 (v/v)). Solutions were prepared from DMSO or MeOH stocks by dilution into buffer in conventional 1 cm PMMA cells. The concentration of the probe used was 0.3 $\mu mol{\cdot}L^{-1}$, and the concentration of the analytes varied from 0 to 32.3 $\mu mol.L^{-1}$. Absorbance spectra of these solutions were recorded over wavelengths 300 – 700 nm using a GBC Cintra 404 spectrometer. Stability constants (*Ks*) were calculated from absorbance changes in the probe using their maximum absorbance (ΔA) by nonlinear regression using online software *Bindfit*[6].

Fluorescence Spectroscopy. Fluorescence of complexes with cholesterol and cholesterol acetate was studied in DMSO and in aqueous medium (H_2O:DMSO; 90:10 (v/v)). The concentration of the salt in complex solutions was 3.125 $nmol.L^{-1}$, whereas, due to high fluorescence intensity, the concentration of FP-5 solution was lowered to 0.39 $\mu mol.L^{-1}$. The ratio of cholesterol and cholesterol acetate, respectively, varied from 1 to 10 equivalents. The changes in fluorescence intensity were measured at the excitation wavelength of 495 nm. Fluorescence spectroscopy studies were carried out using a SCINCO FluoroMate FS-2 spectrometer.

In fluorescence lifetime study, all solutions were excited at 514 nm and the fluorescence spectra were recorded in the wavelength region of 370 to 600 nm using Leica TCS SP8 WLL SMD-FLIM. The fluorescence lifetimes were obtained by the Time Correlated Single Photon Counting (TCSPC) decay method and all decay curves were best fit to double exponential decay.

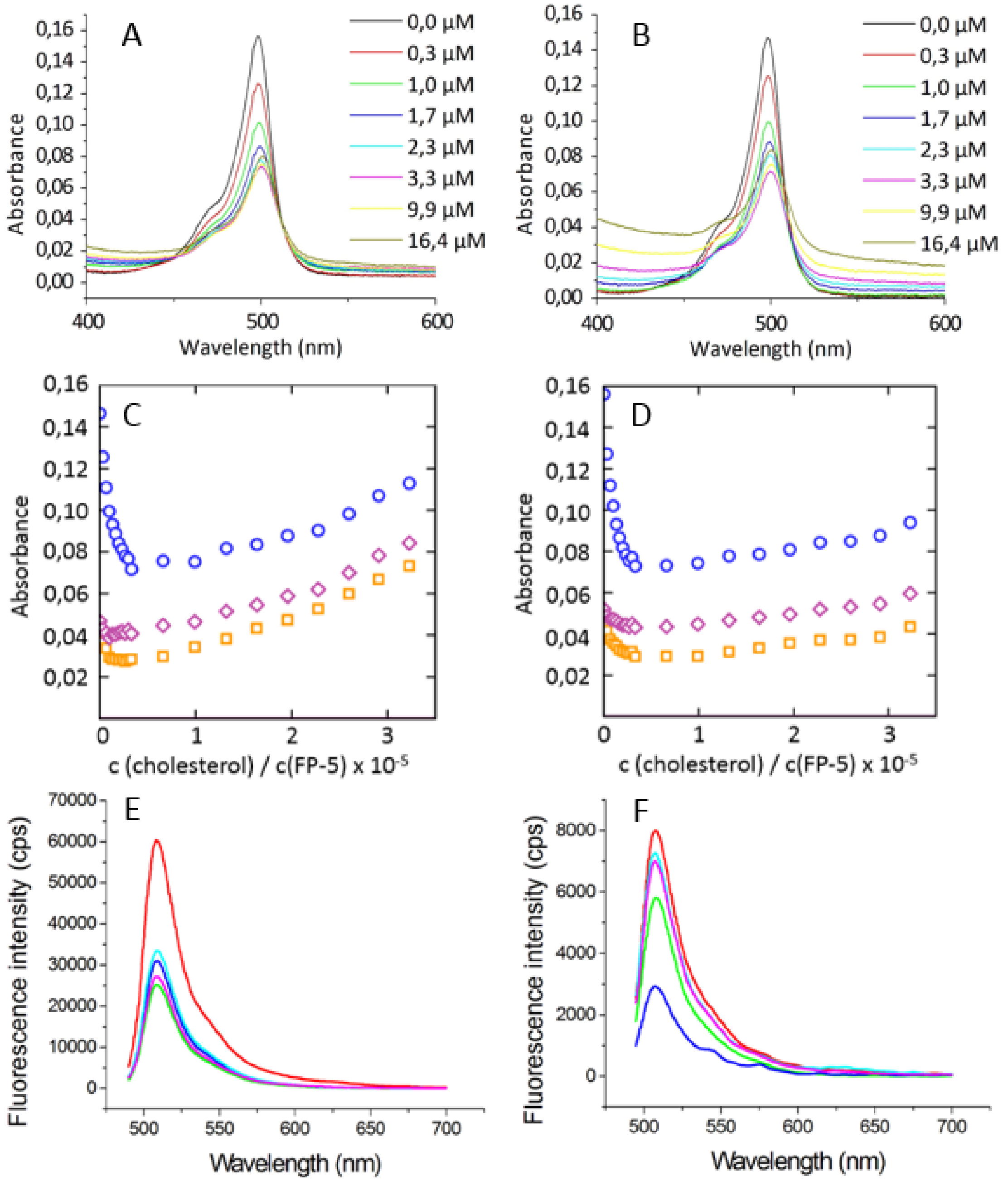

Fig. S6. Interaction of FP-5 with cholesterol and cholesterol acetate measured by UV-VIS and fluorescence spectroscopy. (A, B) Titration of FP-5 with cholesterol (A) and cholesterol acetate (B) in 10% DMSO. (C, D) The absorbance changes measured at several wavelengths ○ 498 nm; ◇ 510 nm; □ 470 nm (C-cholesterol, D-cholesterol acetate). (E, F) FP-5 fluorescence intensity is affected by binding partner and used medium. Complexes of FP-5 with cholesterol and cholesterol acetate in (E) DMSO and (F) H_2O:DMSO; 90:10 (v/v). — FP-5; — FP-5 and cholesterol acetate 1:1; — FP-5 and cholesterol 1:10; — FP-5 and cholesterol 1:1; — FP-5 and cholesterol acetate 1:10.

Table S4. Value of stability constants and stoichiometry of FP-5 complex with cholesterol and cholesterol acetate in aqueous medium H_2O:DMSO; 90:10 (v/v)

Tested ligand	*log K*	Complex stoichiometry (probe : analyte)
Cholesterol	5.99404	1:1
	11.33369	2:1
Cholesterol acetate	6.615559	1:1
	12.84797	2:1

Table S5. Stability constants of FP-5 with cholesterol and cholesterol acetate in PBS buffer with 5% of methanol.

Complex stoichiometry	Cholesterol	Cholesterol acetate
	log K	
1:1	9.5790	11.2847
1:2	13.8575	9.9362

Table S6. Value of fluorescence lifetimes of FP-5 complexes with cholesterol and cholesterol acetate in DMSO.

	FP-5:cholesterol		FP-5:cholesterol acetate		FP-5
Ratio	1:1	1:10	1:1	1:10	
Lifetime (ns)	4.04	4.29	4.20	4.31	4.24
Error (ns)	0.05	0.03	0.03	0.03	0.24

Preparation of liposomes and spectroscopic measurements. F-5-containing liposomes were prepared by passive loading techniques with sonication as mechanical dispersion method described in literature[7-9]. Briefly, soy lecithin was dissolved in water. FP-5 was added to the mixture and sonicated for 10 minutes. Cholesterol and cholesterol acetate was then added to the aqueous medium and absorbance spectra were measured over wavelengths 300 – 700 nm using a GBC Cintra 404 spectrometer. Changes in fluorescence spectra were measured by SCINCO FluoroMate FS-2 spectrometer. The sample was excited at 495 nm.

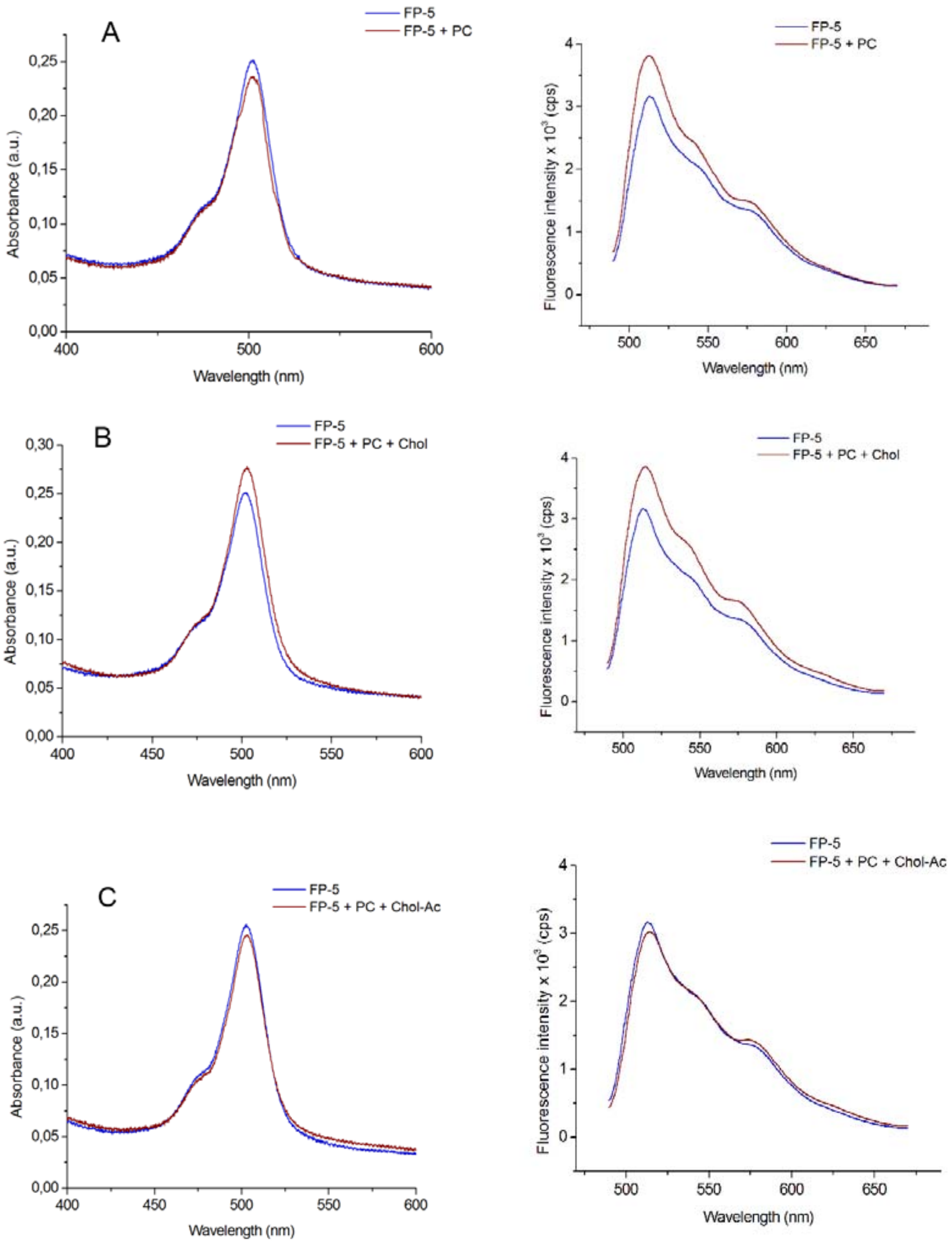

Fig. S7. Absorbance and fluorescence spectra of FP-5 and liposomes containing FP-5, phosphatidylcholine (PC), cholesterol (Chol) or cholesterol acetate(Chol-Ac).

4. Cellular Studies

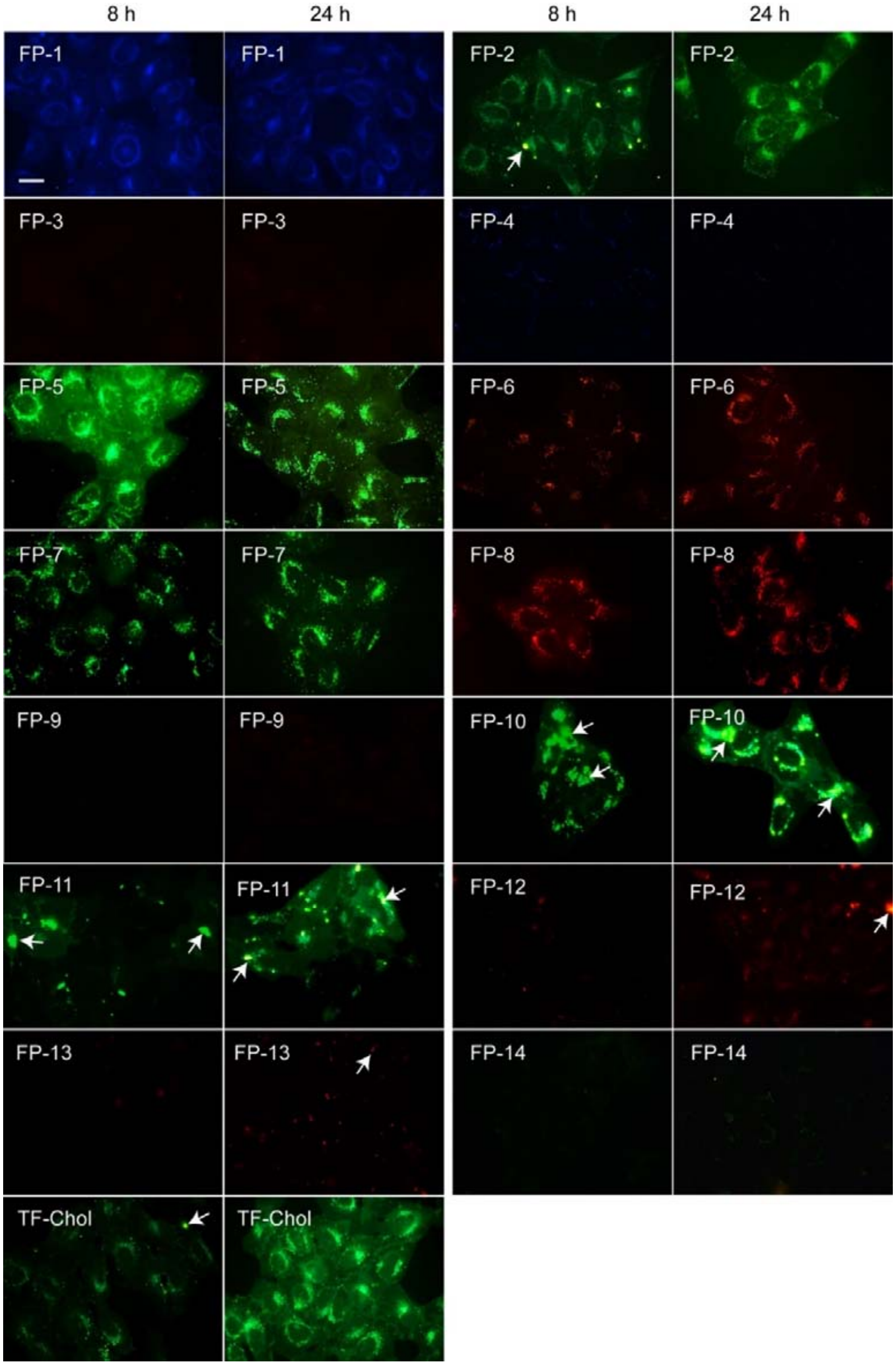

Fig. S8. Live imaging of U-2 OS cells labeled with various heterocyclic sterol probes. Probes FP-5, 7 were directly added to cultivation medium at final concentration 200 nM, while other probes at 500 nM concentration. Cells were incubated with probes for 8 or 24 h, washed and imaged. Commercial TF-Chol (TopFluor-Cholesterol) was included for comparison. Scale bar 10 μm.

Proliferation Study. Cell proliferation assay was conducted using IncuCyte® ZOOM (Essen BioScience, Inc., USA). A2058 and BLM melanoma cells; and PaTu and Panc-1 pancreatic cancer cells were seeded in 96-well plates to the expected target cell density (500 cells·well^{-1}) in six biological replicates. The cells were maintained in 200 µL of complete culture media (DMEM and RPMI with 10% FBS). After 24 h, the seeded cells were treated with 0.2, 1.0, 5.0 or 10.0 µmol·L^{-1} FP-5 added to the culture medium, or did not receive FP-5. Cells were influenced with our compound for 7 days under standard cultivation conditions. The results were plotted as a growth curve.

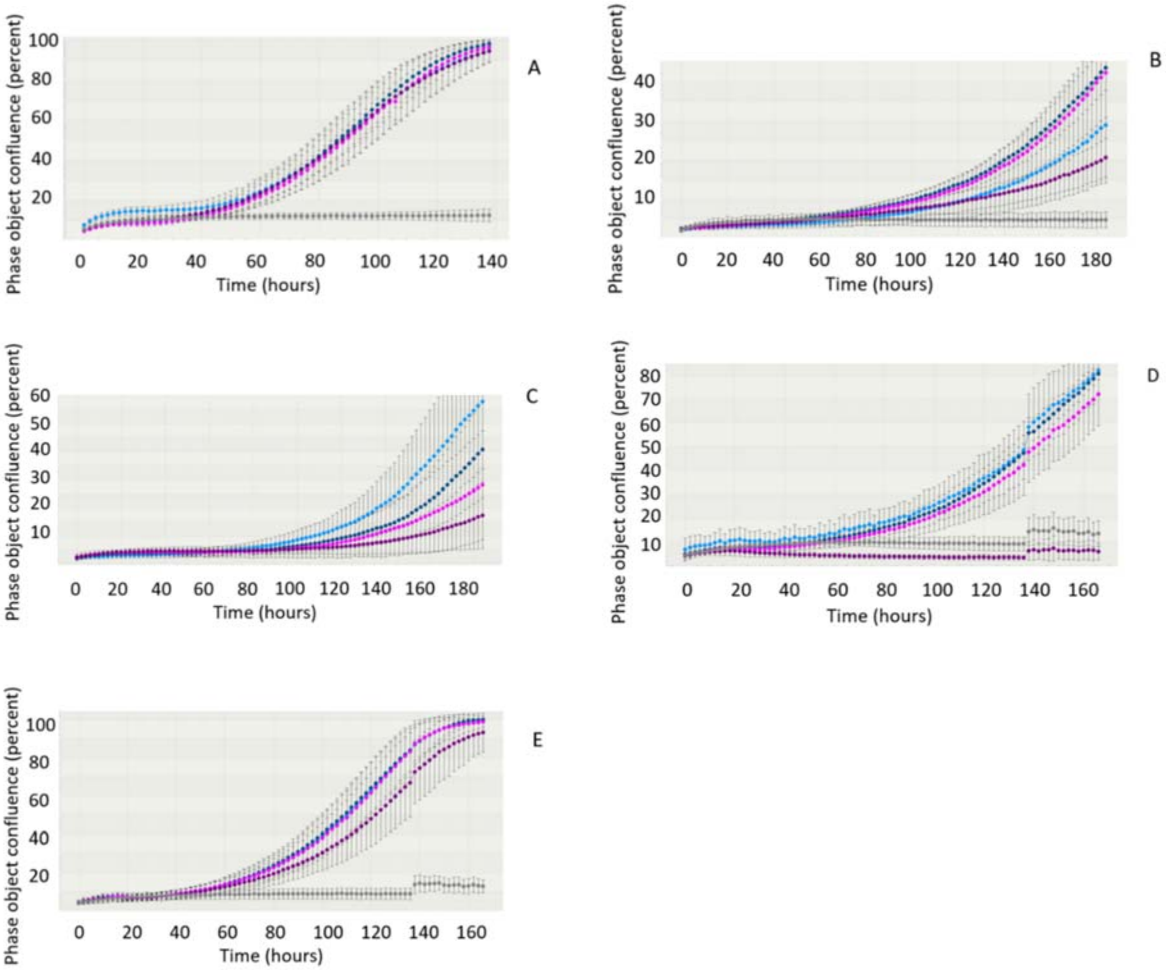

Fig. S9. IncuCyte ZOOM^{tm} proliferation study of FP-5 in U2-OS (A), Panc-1 (B), PaTu (C), A-2058 (D), and BLM cells (E) • control, • 0,2 µM, • 1 µM , • 5 µM , • 10 µM.

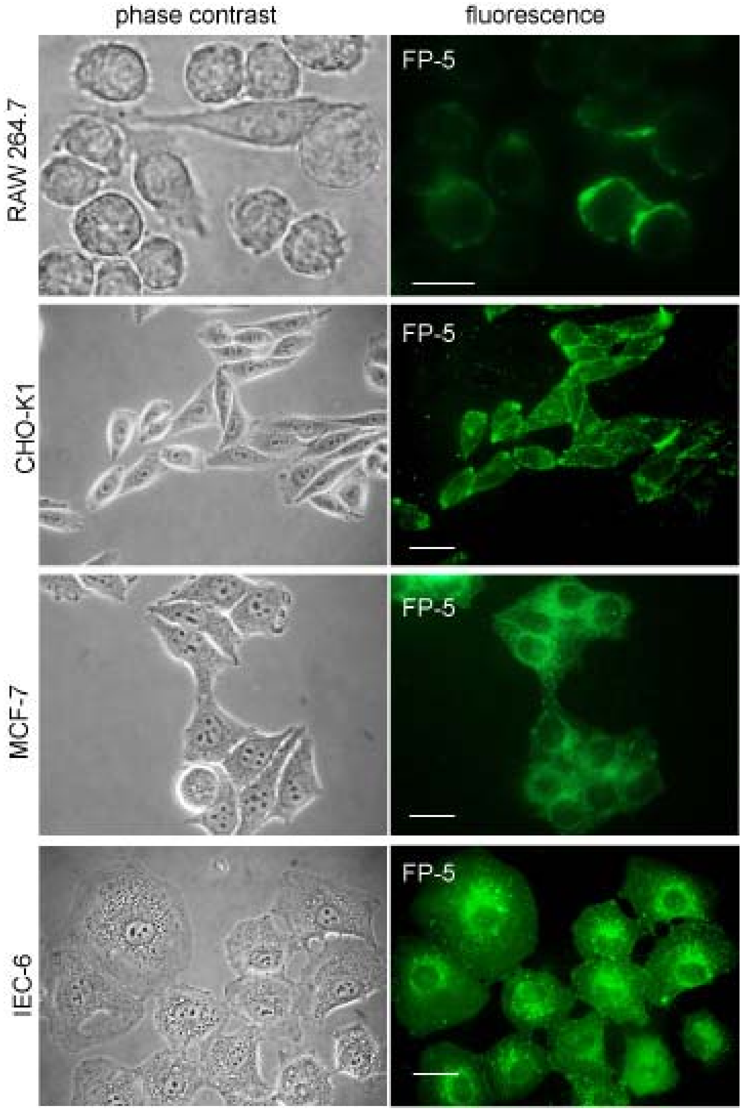

Fig. S10. Labelling various cell lines with FP-5. Raw 264.7 (murine macrophage), CHO-K1 (chinese hamster ovary), 4T1 (mouse mammary carcinoma), MCF-7 (human breast carcinoma), and IEC-6 (normal rat small intestine epithelia) cell lines were labelled for 30 min with 200 nM FP-5 and imaged live. Scale bar 10 μm.

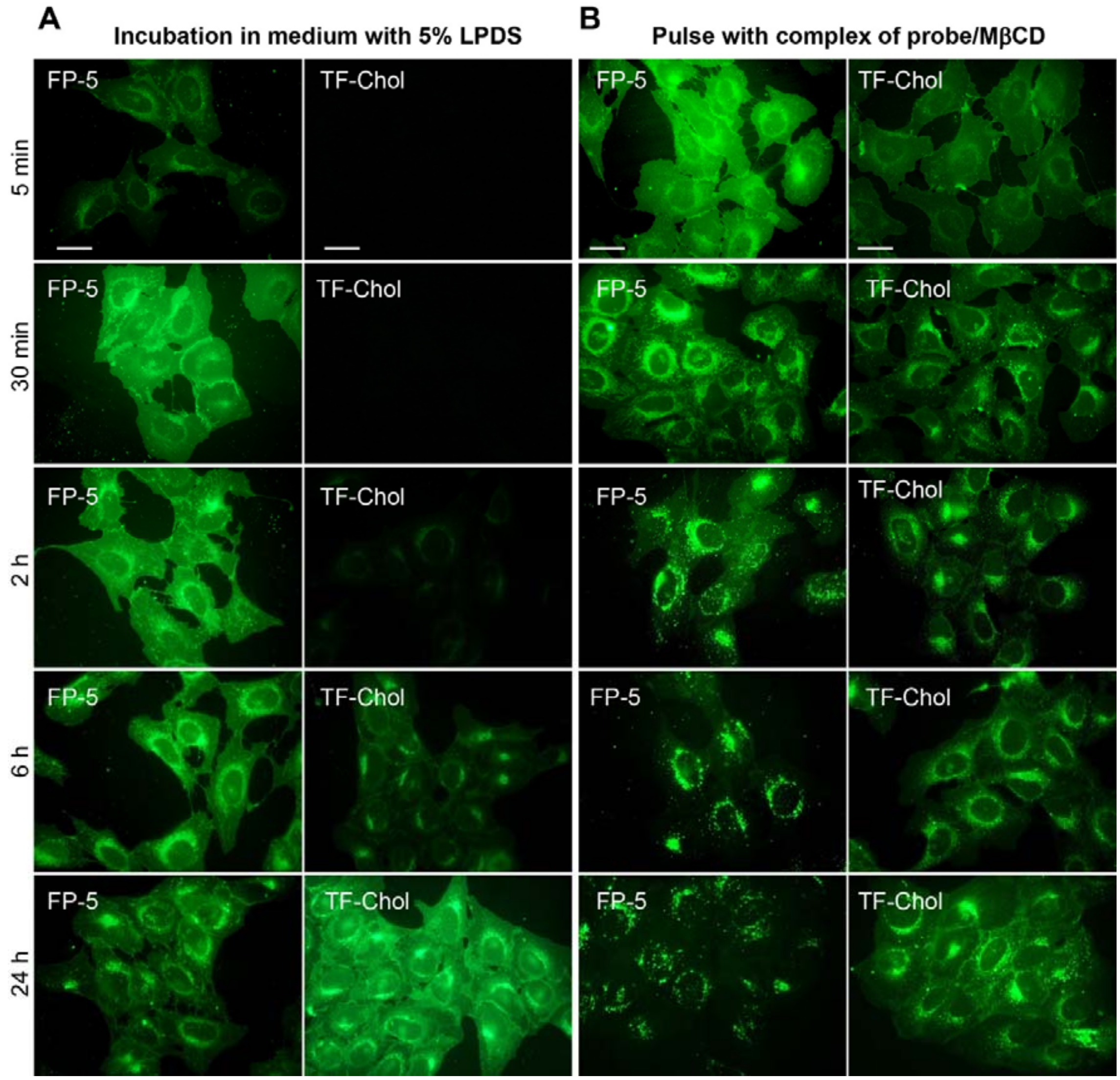

Fig. S11. Kinetics of FP-5 and TF-Chol labeling under various conditions. (A) U-2 OS cells were cultivated overnight in the presence of 5% LPDS followed by incubation with probes at final 0.25 μM concentration. Images were taken at indicated times. (B) Cells were pulsed with complexes of FP-5 (2 μg/ml) or TF-Chol (20 μg/ml) with MβCD (molar ratio 1:10) for 2 min and imaged live at indicated chase times. Scale bar 10 μm.

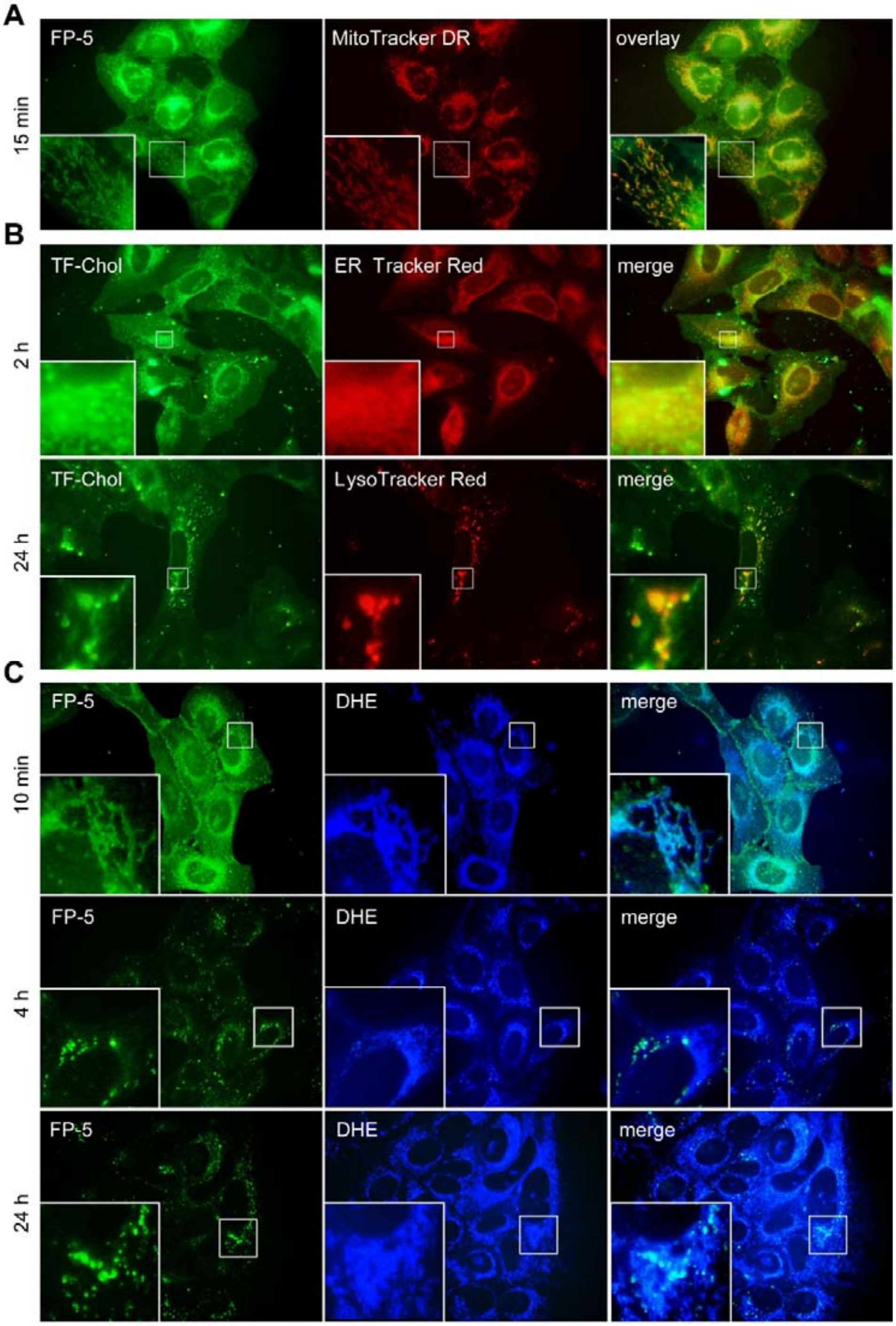

Fig. S12. Localization of FP-5, TF-Chol and DHE in cellular compartments. (A) Transient co-localization of FP-5 and MitoTracker Deep Red in U-2 OS, that were pulsed with complex of FP-5 (2 μg/ml) with MβCD for 2 min, chased for 15 min in medium containing mitochondrial probe MitoTracker Deep Red (80 nM) and imaged. (B) Co-localization of TF-Chol with organelle specific probes in cells pulsed with TF-Chol (20 μg/ml) complexed with MβCD and chased for 2 h or 24 h. For the last 30 min of incubation, organelle probes ER Tracker Red (1μM) and LysoTracker Red (100nM) were added. (C) Co-localization of FP-5 and DHE in U-2 OS cells. Cells pulsed with complexes of DHE (3 mM) and FP-5 (1.5 μM) with MβCD were chased for indicated time at 37 ºC, and imaged alive. Magnifications of the regions indicated by the white boxes are shown on the lower left side.

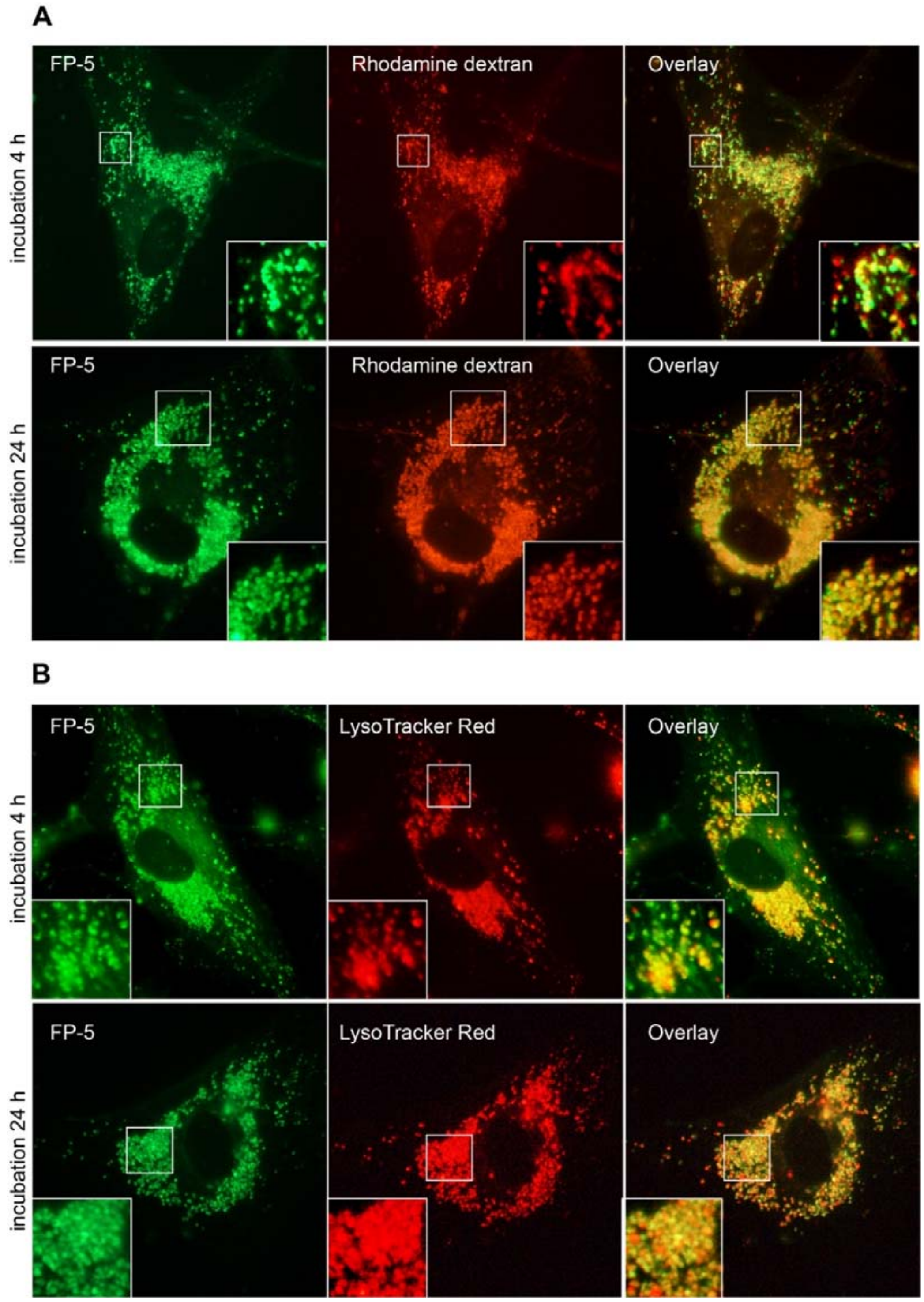

Fig. S13. Co-localization of FP-5 with Rhodamine dextran and LysoTracker Red in NPC1 fibroblasts. Cells were labeled with Rhodamine dextran (1 mg/ml) overnight to label the terminal endocytic compartment, followed by incubation with probe FP-5 (200 nM) for 4 or 24 h. Co-localization was partial at 4 h and complete at 24 h. Similarly, when cells were labeled with FP-5 for the indicated time, followed by a 1 h incubation with LysoTracker Red (20-50 nM), FP-5 was to a considerable extent present in lysosomal compartment within 4 h and almost completely within 24 h.

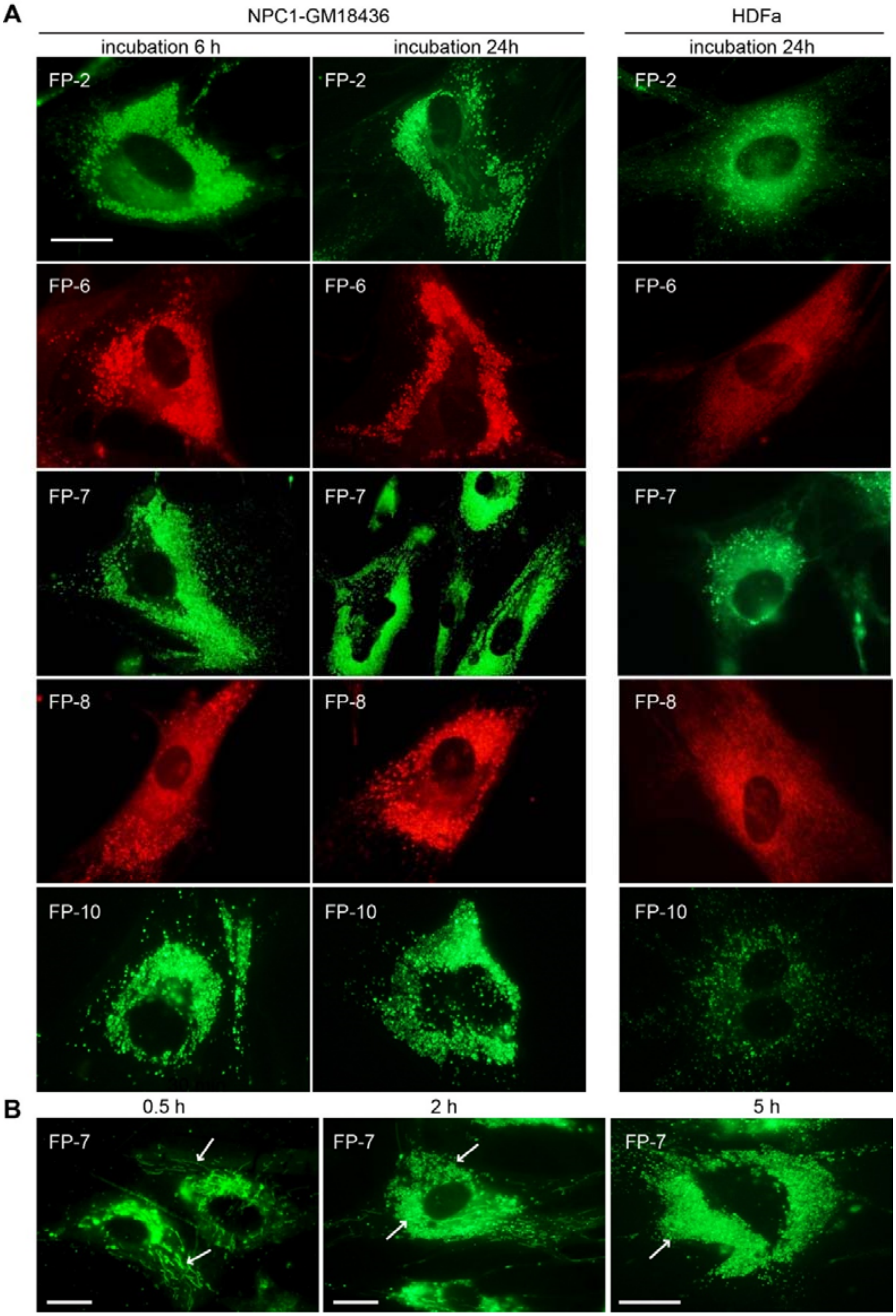

Fig. S14. Labeling of NPC1 fibroblasts with probes FP-2, FP-6, FP-7, FP-8, FP-10. (A) Cells were incubated with 200 nM FP-7 and 1 μM FP-2, FP-6, FP-8 and FP-10 probes for 6 or 24 h and imaged live. Shown for comparison is signal of all used probes in control normal human dermal fibroblasts HDFa. (B) A shift of FP-7 signal from mitochondrial to endo/lysosomal compartments in NPC1 fibroblasts. Cells were pulsed by FP-7/MβCD complex for 2 min and fluorescent signal was imaged live at 30 min, 2 h and 5 h. Arrows point at mitochondrial signal (30 min chase) and endo/lysosomal signal (2 and 5 h chase). Scale bar 10 μm.

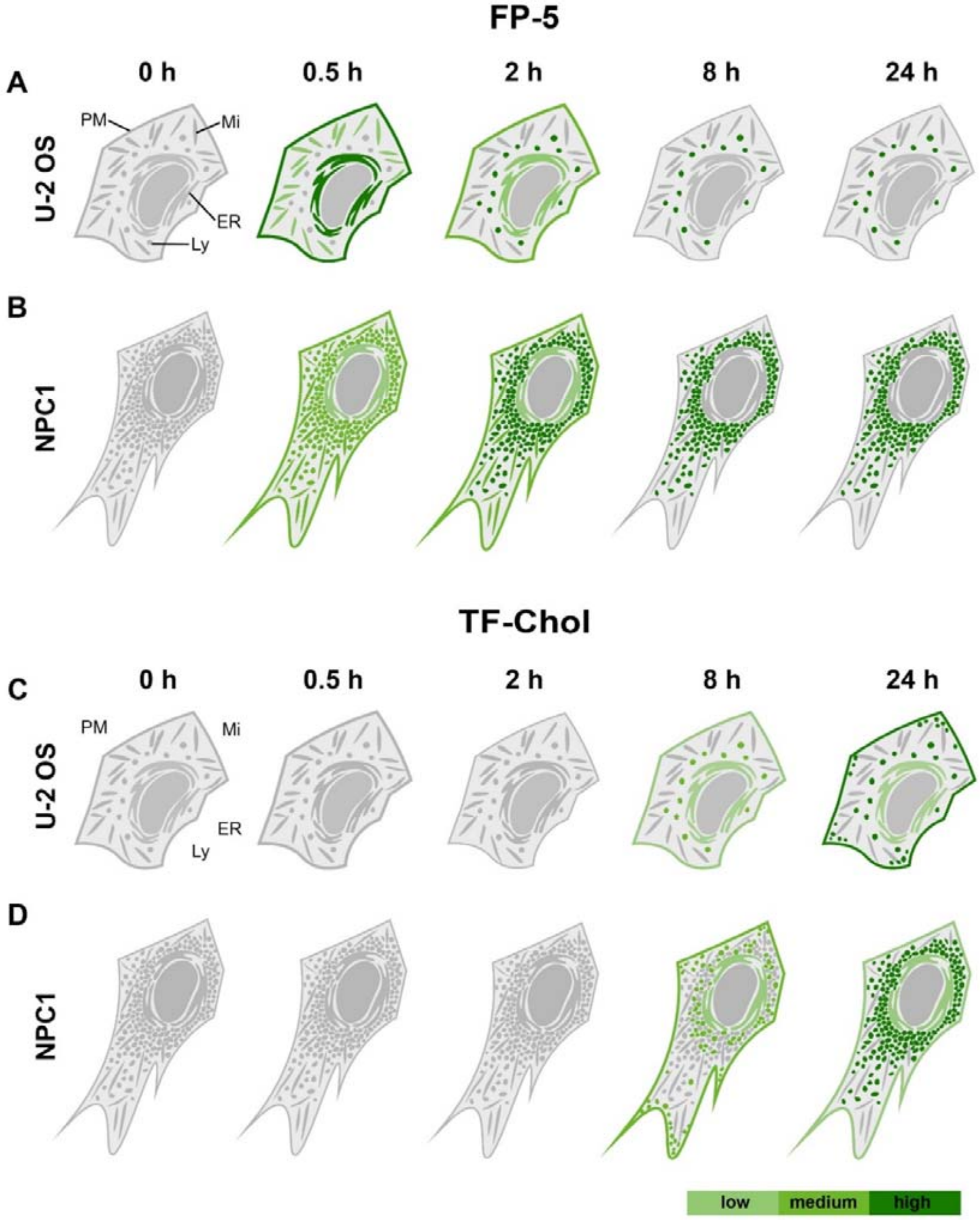

Fig. S15. The comparison of kinetics and compartmentalization of FP-5 and TF-Chol signal in (A) U2-OS cells and (B) in Niemann-Pick fibroblasts following direct addition of probes to cultivation medium.

5. Movie Legends

Movie 1. Time-lapse demonstration of the fluorescence signal emerging on the plasma membranes of U-2 OS cells during pulse with FP-5/MβCD (time 0 – 1.5 min). Frame rate interval for acquisition was set on 10 seconds.

Movie 2. Time-lapse showing dynamic redistribution of FP-5 signal on cellular membranes and inracellular structures during a 5 -10 min interval after pulse. Arrowheads point at gradual emergence of FP-5 signal in endoplasmic reticulum. Frame rate interval for acquisition was set on 10 seconds.

Movie 3. Time-lapse showing dynamic redistribution of FP-5 signal on cellular membranes and intracellular structures during a 20-25 min interval after pulse. Arrowhead point at labeling and movement of endo/lysosomes. Frame rate interval for acquisition was set at 10 seconds.

6. References

1. Wang, D. C. *et al.* Carboxyl BODIPY Dyes from Bicarboxylic Anhydrides: One-Pot Preparation, Spectral Properties, Photostability, and Biolabeling. *J Org Chem* **74**, 7675-7683, doi:10.1021/jo901149y (2009).
2. Malachowska-Ugarte, M. *et al.* Brassinosteroid-BODIPY conjugates: Design, synthesis, and properties. *Steroids* **102**, 53-59, doi:http://dx.doi.org/10.1016/j.steroids.2015.07.002 (2015).
3. Palatinus, L. & Chapuis, G. SUPERFLIP - a computer program for the solution of crystal structures by charge flipping in arbitrary dimensions. *J Appl Crystallogr* **40**, 786-790, doi:10.1107/S0021889807029238 (2007).
4. Petricek, V., Dusek, M. & Palatinus, L. Crystallographic Computing System JANA2006: General features. *Z Krist-Cryst Mater* **229**, 345-352, doi:10.1515/zkri-2014-1737 (2014).
5. Diamond (Crystal and Molecular Structure Visualization Crystal Impact - Dr. H. Putz & Dr. K. Brandenburg GbR, Kreuzherrenstr. 102, 53227 Bonn, Germany http://www.crystalimpact.com/diamond.

6a. Thordarson, P. Determining association constants from titration experiments in supramolecular chemistry *Chem Soc Rev* **40**, 1305– 1323, DOI: 10.1039/C0CS00062K, (2011)

6b. www.supramolecular.org (accessed May 10, 2018).

7. Dua, J.S., Rana, A.C., Bhandari, A.K. Liposome: Methods of preparation and application. IJPSR **3**,14-20 (2012)
8. Szoka, F. Comparative properties and methods of preparation of lipid vesicles (liposomes). *Ann. Rev. Biophys. Bioeng* **9**, 467-508 (1980)
9. Cho, N.-J., Hwang, L.Y., Solandt, J.J., Frank, C.W. Comparison of extruded and sonicated vesicles for planar bilayer self-assembly. *Materials* **6**, 3294-3308, doi:10.3390/ma6083294 (2013)
